# A Critical Review of the Propagation Models Employed in LoRa Systems

**DOI:** 10.3390/s24123877

**Published:** 2024-06-15

**Authors:** Joaquim Amândio Azevedo, Fábio Mendonça

**Affiliations:** 1 Center for Research in Mathematics and Applications (CIMA), Faculty of Exact Sciences and Engineering, University of Madeira, 9020-105 Funchal, Portugal; 2 Faculty of Exact Sciences and Engineering, University of Madeira, 9020-105 Funchal, Portugal; fabioruben@staff.uma.pt; 3 Interactive Technologies Institute (ITI/LARSyS and ARDITI), Edif. Madeira Tecnopolo, Caminho da Penteada Piso-2, 9020-105 Funchal, Portugal

**Keywords:** LoRa, propagation models, path loss prediction, outdoor environments, indoor environments, RSSI, SNR, ESP, measurements, environmental parameters

## Abstract

LoRa systems are emerging as a promising technology for wireless sensor networks due to their exceptional range and low power consumption. The successful deployment of LoRa networks relies on accurate propagation models to facilitate effective network planning. Therefore, this review explores the landscape of propagation models supporting LoRa networks. Specifically, we examine empirical propagation models commonly employed in communication systems, assessing their applicability across various environments such as outdoor, indoor, and within vegetation. Our investigation underscores the prevalence of logarithmic decay in most empirical models. In addition, we survey the relationship between model parameters and environmental factors, clearing their nuanced interplay. Analyzing published measurement results, we extract the log-distance model parameters to decipher environmental influences comprehensively. Drawing insights from published measurement results for LoRa, we compare them with the model’s outcomes, highlighting successes and limitations. We additionally explore the application of multi-slope models to LoRa measurements to evaluate its effectiveness in enhancing the accuracy of path loss prediction. Finally, we propose new lines for future research in propagation modelling to improve empirical models.

## 1. Introduction

LoRa technology has stood out in the domain of wireless sensor networks (WSNs) due to its long-range capabilities [1]. This technology enables low-power, low-cost, and reliable data transmission, offering a versatile and responsive framework for diverse applications ranging from agriculture and environmental monitoring to smart cities and industrial automation [2,3,4,5,6,7,8]. Deploying a sensor network in real-world environments requires accurate estimations of the received signal. Consequently, modelling the environmental impact on LoRa communications becomes paramount [9].

Propagation models usually provide two types of parameters: large-scale path loss and small-scale fading statistics [10,11]. Large-scale fading represents the average path loss and shadowing over large distances. The signal fluctuation is affected primarily by the presence of hills, forests, and buildings between the transmitter and the receiver. Small-scale fading refers to the dramatic changes in the received signal because of small changes in position. A stochastic process typically characterizes the statistical description of the amplitude distribution of the received signal. Some statistical models for small-scale fading are the Rayleigh distribution, the Rican distribution, the log-normal fading model, or the Nakagami model [10].

In the literature, authors classify propagation models in various ways. Iskander et al. [12] classify the path loss models as empirical, site-specific, and theoretical. Empirical models rely on intensive measurements. While simple, they often exhibit low accuracy when employed in environments other than data collection locations. Site-specific models are derived from a detailed understanding of the environment. These models provide high-accuracy predictions but demand extensive databases containing environmental parameters. Theoretical models use the theory of idealized electromagnetic propagation. Sarkar et al. [10] classified the models as empirical or statistical and deterministic or site-specific. Theoretical models fall outside the scope of model classification. Phillips et al. presented a survey classifying propagation models in seven categories [13]. The first group is theoretical or foundational models. These models are purely analytical and derived from the theory of idealized electromagnetic propagation. The second group is basic models whose input parameters are the distance, the carrier frequency, and the transmitter and the receiver heights. Various propagation models applied to communication systems fall into this category. The third type is terrain models. These models are similar to the basic models but also attempt to compute diffraction losses due to the presence of obstacles. The fourth category, classified as supplementary models, comprises those unable to function independently. In this case, researchers adjust existing models, such as employing different frequency coverage, addressing obstructions, or incorporating directivity considerations. The fifth group considers stochastic fading models that add a random variable to account for additional fading. The sixth category is many-ray models, commonly known as ray-tracing models. The final group is active measurement models. In the application to unfamiliar environments, these models rely on measurements to enhance their accuracy. For comparison, the work presented in [14] includes a table with the typical propagation model classifications. Several classifications only consider two categories, with some further subdivided into different groups. As we may observe, there is no unanimity among authors in the propagation model classification.

The survey outlined in this study will examine three types of environments: outdoor, indoor, and within vegetation. This categorization is motivated by the necessity to align with prevalent propagation models employed in communication systems for path loss characterization. Examples of models that address other types of propagation media, such as indoor–outdoor propagation [15] or underground propagation [16], are beyond the scope of the objectives of this study.

Researchers are proposing several conventional propagation models to estimate the received signal in LoRa systems. For outdoor environments, numerous studies employed the log-distance model to predict path loss through the curve fitting of measurements [2,3,17,18,19,20,21,22,23,24,25,26,27,28,29,30] or for model evaluation [31,32,33,34,35,36]. All works of the first group use active measurements on the proposed model. Although most proposed log-distance models are single-slope, some authors show that a dual-slope log-distance model can improve the model’s accuracy [23,27,34]. For instance, Abdelfadeel et al. [23] proposed a two-component model, one for distances below 400 m and another for distances from 400 m and 1 km. The Okumura–Hata model has also been considered in various works for predicting path loss [7,37,38,39,40,41,42,43] or model evaluation [31,32,33,34,35,36,44,45,46,47,48,49,50,51,52]. COST 231-Hata is utilized in [32,34,36,46,48,50], the COST–Walfisch–Ikegami model is used in [33,46,48,51], the 3GPP model is used in [31,32,33,48,53], and the SUI model is used in [33,35,48,50]. We also found the use of the Lee model [54], the Irregular Terrain With Obstructions Model (ITWOM) [42,55], the ITU-R P 526 model [39], the ITU-R P 1812 model [56,57], the Irregular Terrain Model (ITM) [47,58,59], the Ericsson model [33,35,48], the Egli model [5,56], the ECC33 model [49], and the UFPA model [6]. Some works also include variations of the well-known models. For example, González-Palacio et al. [60] proposed enhancing the log-distance model by including terms related to the environmental parameters. Furthermore, Santana et al. [61] developed a model centred on the log-distance, adjusting the path loss exponent based on measured data.

Regarding deterministic models for outdoor propagation environments, very few works have focused on estimating path loss. Froiz-Míguez et al. [62] employed a 3D ray model for planning purposes. The simulation results show the received signal estimation for an area of 100 × 200 m^2^. Dão et al. [63] predicted the received signal using the Oscillator Finite-Difference Time-Domain (O-FDTD) method. The authors state that this model may become unsustainable for LoRa maps much larger than 600 × 600 m^2^. Machine learning also provided tools to assist the studies of received signal prediction in LoRa [45,64,65,66,67,68].

For indoor environments, the propagation models commonly employed in LoRa studies include the log-distance [69,70,71,72,73], the ITUR P1238 [69,70,74], the Montley–Keanan [32,69,70,73], and the COST 231 Multi-Wall [32,69,70,71,73]. Studies on propagation have also proposed models of ray tracing model [69,73], the 3GPP indoor [32], the Indoor Dominant Path (IDP) [75], and linear decaying [76]. The works presented in [77,78] introduce machine learning techniques on LoRa indoor propagation studies.

There are several conventional models designed specifically for environments within vegetation. Typically, researchers formulated these models to predict excess attenuation caused by vegetation. LoRa propagation studies within vegetation considered the Weissberger model [79,80,81], the ITU-R P.833 model [8,79,80,81,82], the FITU-R model [8,79,80,81,82], the COST 235 model [8,79,80,81,82], and the LITU model [8,81,82]. Some authors employed these models by contrasting them with measurements taken within vegetation to choose the best one. Another approach involved comparing the models after optimizing their parameters to align with the measured data.

The suitability of applying the Okumura–Hata model in environments within vegetation is presented in studies [83,84]. Ferreira et al. [85] adjusted the log-distance model with measurements acquired on a university campus to obtain a propagation model in forested environments for LoRa. Wu et al. [86] proposed formulas for the parameters of the log-distance model, which incorporate constants associated with vegetation. Anzum [87] presents a study that quantifies the excess attenuation introduced by palm oil trees for propagation through the trunk, the canopy, and the treetop. The work presented in [88] applies the COST231-multiwall model to a palm oil plantation, replacing the number of walls with the number of trunks and canopies. Myagmardulam et al. [89] studied the correlation between the received signal and the sky view factor in a forested area. The works presented in [8,84,90] employ machine learning techniques to predict path loss in the presence of vegetation.

Based on the current research, we may classify the propagation models for LoRA into three categories: empirical models, determinist models, and active measurement models. This study reveals that the majority of the proposed models are empirical. There are very few works focused on deterministic models. This fact may stem from the need for extensive databases containing environmental parameters, especially considering the wide range of LoRa. Active measurement models include curve fitting of existing empirical models and models based on machine learning techniques.

Rather than systematically searching a specific subfield or examining all published articles that examine propagation models for LoRa, we intended to review the most relevant pathways by examining well-known literature that operates as a reference for the field and extrapolate conclusions based on these works. Therefore, this work aims to perform a critical review to achieve the following objectives:

- Review the propagation models applied to LoRa systems;
- Review the empirical propagation models designed to support communication system projects in outdoor, indoor, and vegetation environments, potentially beneficial for LoRa;
- Characterize the parameters outlined for each empirical propagation model;
- Extract the relevant information from the measurements presented in published works to understand propagation phenomena;
- Analyze propagation results of LoRa systems;
- Assess the performance of the studied propagation models using LoRa measurements;
- Evaluate the potential of a multi-slope model for path loss prediction in LoRa systems.

This paper is structured into six sections. Section 1 provides an overview of commonly employed propagation models in LoRa technology. Section 2 describes the empirical propagation models, dissecting them based on their primary parameters. In Section 3, we explore active measurement propagation models, exploring their potential as complementary or alternative methods for propagation analysis. Section 4 shows a critical analysis of propagation measurements utilized in the reviewed literature. Section 5 presents a complementary examination of propagation within LoRa systems. Finally, Section 6 explains the main findings and concludes the article.

## 2. Empirical Propagation Models

Both theoretical and measurements indicate an average received signal decaying logarithmically with distance plus a log-normal distribution about the mean value [91]. In this way, the log-normal path loss model is given by, in dB [92]

(1) $$PL\left(d\right)=PL\left(d_{0}\right)+10n{log}_{10}\left(\frac{d}{d_{0}}\right)+X_{\sigma }$$

where $PL\left(d_{0}\right)$ is the mean path loss given at a distance $d_{0}$ in dB, *n* is the path loss exponent, and $X_{\sigma }$ is a zero mean Gaussian distributed random variable with standard deviation σ in dB.

Rappaport et al. [93] defend that *n* has a physical meaning when $PL\left(d_{0}\right)$ is the free space path loss at the reference distance $d_{0}$. In that case, (1) is defined as a Close-In (CI) model [94]. However, many authors use this model to fit the measured data. In this situation, $PL\left(d_{0}\right)$ can be different from that of free space. To distinguish both cases, some authors propose the floating-intercept (FI) model, given by

(2) $$PL\left(d\right)=\alpha +10\beta {log}_{10}\left(d\right)+X_{\sigma }$$

where α is the intercept in dB and β is the slope of the line with the distance in a logarithmic scale. By equating the two expressions and setting β=n, we obtain

(3) $$\alpha =PL\left(d_{0}\right)-10n{log}_{10}\left(d_{0}\right)$$

This result suggests that given α and β, we can obtain the reference distance, $d_{0}$, where both models are equivalent. The distance where the floating-intercept (FI) model crosses the free-space condition is

(4) $$d_{0}={\left[{\left(\frac{4\pi }{\lambda }\right)}^{2}\times 10^{-\frac{\alpha }{10}}\right]}^{1/(\beta -2)},\;\beta >2\;$$

with λ indicating the wavelength.

We will employ the log-distance model as the foundational equation for defining empirical propagation models. Emphasizing the parameters of these models is essential to facilitate meaningful comparisons among them. By transforming each model into the format outlined in Equation (2), we will extract expressions for α and β specific for each empirical model. After identifying the main parameters related to the propagation environment, α will take the following form:

(5) $$\alpha ={PL}_{FS}\left(1\;m\right)+K_{\alpha }+P_{1}\left(f\right)+P_{2}\left(h_{B},h_{T}\right)+\cdots \;$$

where ${PL}_{FS}\left(1\;m\right)$ is the free space path loss at a distance of one meter in dB, $K_{\alpha }$ is a constant defined in each propagation model for the α parameter in dB, and $P_{1}\left(f\right)$ is the attenuation relative to frequency, obtained by subtracting $20{log}_{10}\left(f\right)$ in dB. The other terms are losses related to the parameters given in each function, with $h_{B}$ representing the height of the base station antenna and $h_{T}$ indicating the height of the terminal device antenna. The parameter β will take the following form:

(6) $$\beta =K_{\beta }+F_{1}\left(f\right)+F_{2}\left(h_{B},h_{T}\right)+\cdots$$

The distances will be in meters and the frequency in MHz.

Let us describe the procedure for the Okumura–Hata model [95,96]. For urban environments, the model is given by

(7) $${PL}_{urban}=69.55+26.16{log}_{10}\left(f\right)-13.82{log}_{10}\left(h_{B}\right)+\left[44.9-6.55{log}_{10}\left(h_{B}\right)\right]{log}_{10}\left(d\right)-a(h_{T})$$

with *f* in MHz, *d* in km, $h_{B}$ and $h_{T}$ in m, and $a(h_{T}$) is given for medium-small cities by

(8) $$a\left(h_{T}\right)=\left[1.1{log}_{10}\left(f\right)-0.7\right]h_{T}-\left[1.56{log}_{10}\left(f\right)-0.8\right]$$

After converting the distance to meters and manipulating (7) to identify the individual components, we obtain

(9) $$\begin{array}{lll} {PL}_{urban} & =69.55 & +26.16{log}_{10}\left(f\right)-13.82{log}_{10}\left(h_{B}\right)+\left[44.9-6.55{log}_{10}\left(h_{B}\right)\right]{log}_{10}\left(\frac{d}{1000}\right)\\ & & -\left[1.1{log}_{10}\left(f\right)-0.7\right]h_{T}+1.56{log}_{10}\left(f\right)-0.8+{PL}_{FS}\left(1\;m\right)-20{log}_{10}\left(\frac{4\pi f\times 10^{6}}{3\times 10^{8}}\right)\\ & ={PL}_{FS}\left(1\;m\right) & -38.39+7.72{log}_{10}\left(f\right)+5.83{log}_{10}\left(h_{B}\right)-\left[1.1{log}_{10}\left(f\right)-0.7\right]h_{T}\\ & & +10\left[4.49-0.655{log}_{10}\left(h_{B}\right)\right]{log}_{10}\left(d\right) \end{array}$$

This form allows us to identify each term in Equations (5) and (6).

### 2.1. Outdoor Environments

We identified empirical propagation models for outdoor environments and converted the expressions into a log-distance form. Table 1 shows the results for two simple basic models. The Egli model was developed from measurements conducted in New York and New Jersey [97,98]. As we may observe from the third column of Table 1, the component α only varies with the antenna heights. For this model, β is constant. The Lee model was formulated based on a series of measurements conducted in the USA, specifically at a frequency of 900 MHz [99,100]. The third column of Table 1 shows the results. *G_B_* and *G_T_* are the antenna gains in dB, and *m* is a frequency adjustment factor, 2 ≤ *m* ≤ 3. $L_{0}$ is the path loss at 1 km, and γ is the slope of the path loss curve in dB/decade. We incorporated the antenna gains into the parameter $K_{\alpha }$, as it is common practice to exclude the transmitted power and antenna gains from the loss computation. Table 2 provides values for the parameters $L_{0}$ and γ.

Table 3 presents the outcomes of the Okumura–Hata model [99,100], designed for large urban, medium–small urban, suburban, and rural environments. The distance range remains applicable within 1 km to 20 km, with base station antenna heights ranging from 30 m to 200 m and terminal antenna heights from 1 m to 20 m. Frequency coverage extends from 200 MHz to 1500 MHz. Because propagation model evaluation will be specific for frequency bands of LoRa, $P_{1}\left(h_{B},h_{T}\right)$ incorporates products with frequency and antenna height terms. From column three of Table 3, $K_{\alpha }$ decreases from urban to rural areas. For medium–small cities, suburban areas, and rural areas, the α component varies solely by frequency, remaining constant across antenna heights. The β parameter remains invariant across all environments.

The widespread popularity of the Okumura–Hata model has led to the development of numerous extensions and corrections aimed at refining its accuracy and applicability. An example is the Ericsson model [101]. Table 4 shows the parameters obtained for this model. Parameters $a_{0}$, $a_{1}$, $a_{2}$, and $a_{3}$ have the default values of 36.2, 30.2, −12, and 0.1, respectively. The COST 231-Hata model is another extension of the Okumura–Hata model. It aims to extend the frequency band to 2000 MHz [102] and has applications in urban and suburban/rural environments. The Stanford University Interim (SUI) model follows the form represented in Equation (1) [103]. The reference distance is $d_{0}=100\;m$. This model also extends the Okumura–Hata frequency range up to 2000 MHz. The model is valid for antenna heights of the base station between 10 and 80 m and defines three types of environments. The type A environment corresponds to the maximum path loss category, characterized by hilly terrain with moderate-to-heavy tree densities. Flat terrains with moderate to heavy tree densities or hilly terrains with light tree densities characterize type B environments. Type C environments, representing the minimum path loss category, are applied to flat terrain with light tree densities. Table 4 shows the results obtained for the SUI model.

The Electronic Communication Committee (ECC) developed the ECC 33 model that predicts the path loss for frequencies greater than 3 GHz [104]. The model contains a term of the form ${\left[{log}_{10}\left(d\right)\right]}^{2}$. For this reason, the model does not conform to a linear trend when plotted against distance on a logarithmic scale. Table 4 shows the log-distance representation for this model. As we may observe, the β component associated with $h_{B}$ exhibits distance dependency. Considering the values of $h_{B}$ between 30 m and 200 m and distances from 1 km to 20 km, we developed an approximation for the second term of (2), giving

(10) $$\begin{array}{ll} 10\beta {log}_{10}\left(d\right) & =10\left[2.98+0.58\left[{6-log}_{10}\left(d\right)\right]{log}_{10}\left(\frac{h_{B}}{200}\right)\right]{log}_{10}\left(d\right)\\ & ≅82.18{log}_{10}\left(\frac{h_{B}}{200}\right)+10\left[2.98-0.9{log}_{10}\left(\frac{h_{B}}{200}\right)\right]{log}_{10}\left(d\right) \end{array}$$

The first term of this expression is added to α and the new approximated β is $2.98-0.9{log}_{10}(h_{B}/200)$. The maximum error for this approximation occurs for $h_{B}=30\;m$, giving a mean absolute error of 0.41 dB.

The previous empirical models may have reduced accuracy as they only incorporate frequency, antenna heights, and distance as input parameters. The next group of models also includes parameters related to the environment. Specifically, the Edwards–Durkin model calculates the total loss over irregular terrain by the expression $\max\left({PL}_{FS},{PL}_{PE}\right)+L_{D}$ [98,105], where max is the maximum of its arguments, ${PL}_{FS}$ is the free-space path loss model, ${PL}_{PE}$ is the plane-earth path loss model and $L_{D}$ is the diffraction due to the irregular terrain in dB. Table 5 shows the log-distance representation. The free-space model is applicable up to $d=4\pi h_{B}h_{T}/\lambda$ and the plane-earth model is valid above this distance. We may use the knife-edge diffraction loss, the Eperstein–Peterson method [106], or the Longley–Rice model [107] to compute ${PL}_{D}.$ The component $P_{3}\left(\upsilon \right)$ represents the dependence of the diffraction parameter υ.

The Blomquist–Ladell model [108] addresses identical loss types as the Edwards–Durkin model but employs a distinct methodology in combining them,

(11) $$PL(d)={PL}_{FS}+\sqrt{{\left({PL}_{PE}^{\prime }-{PL}_{FS}\right)}^{2}+L_{D}^{2}}$$

where ${PL}_{PE}^{\prime }$ is a modified plane earth loss. The Allsebrook–Parsons model [109] is an extension of the Blomquist–Laddell model that adds a loss due to buildings,

(12) $$PL(d)={PL}_{FS}+\sqrt{{\left({PL}_{PE}^{\prime }-{PL}_{FS}\right)}^{2}+L_{D}^{2}}+L_{B}+\gamma$$

where $L_{B}$ is an additional loss due to buildings and γ is a UHF correction factor.

The second example described in Table 5 is the Ikegami model [110]. This model assumes free space propagation, augmented by an additional term designed to accommodate the signal behaviour within a street environment. H is building height, *W* is street width, and ϕ is the angle of signal arrival relative to the street axis. The next propagation model shown in Table 5 is the Walfisch–Bertoni model [111]. *h* is the average building height, and D is the distance between building centres. We ignored the term that accounts for the curvature of the Earth. The COST 231-Walfisch–Ikegami model [102] has a version for a street canyon with Line-Of-Sight (LOS). A representation by Equation (2) considers $K_{\alpha }=-7.84\;dB$ and β=2.6 and is valid for distances above 20 m. For Non-Line-Of-Sight (NLOS), the model is given by

(13) $$PL(d)=\left\{\begin{array}{cc} {PL}_{FS}+L_{rts}+L_{msd} & ,L_{rts}+L_{msd}\geq 0\\ {PL}_{FS} & {,L}_{rts}+L_{msd}<0 \end{array}\right.$$

with

(14) $$L_{rts}=-16.9-10{log}_{10}\left(W\right)+10{log}_{10}\left(f\right)+20{log}_{10}\left({h-h}_{T}\right)+L_{ori}$$

(15) $$L_{msd}=L_{bsh}+k_{a}+k_{d}10{log}_{10}\left(\frac{d}{1000}\right)+k_{f}{log}_{10}\left(f\right)-9{log}_{10}\left(D\right)$$

(16) $$L_{ori}=\left\{\begin{array}{cc} -10+0.325\phi & 0^{{}^{\circ}}\leq \phi <35^{{}^{\circ}}\\ 2.5+0.075(\phi -35) & 35^{{}^{\circ}}\leq \phi <55^{{}^{\circ}}\\ 4.0-0.114(\phi -55) & 55^{{}^{\circ}}\leq \phi <90^{{}^{\circ}} \end{array}\right.$$

(17) $$L_{bsh}=\left\{\begin{array}{cc} -18{log}_{10}\left(1+h_{B}-h\right) & ,h_{B}>h\\ 0 & ,h_{B}\leq h \end{array}\right.$$

(18) $$k_{a}=\left\{\begin{array}{cc} 54 & h_{B}>h\\ 54-0.8{(h}_{B}-h) & h_{B}\leq h\;and\;d\geq 500\;m\;\\ 54-0.8{(h}_{B}-h)\frac{d}{500} & h_{B}\leq h\;and\;d<500\;m \end{array}\right.$$

(19) $$k_{d}=\left\{\begin{array}{cc} 18 & ,h_{B}>h\\ 18-15\left(\frac{h_{B}-h}{h}\right) & ,h_{B}\leq h \end{array}\right.$$

(20) $$k_{f}=\left\{\begin{array}{cc} -4+0.7\left(\frac{f}{925}-1\right) & ,\;medium-sized\;cities\;and\;suburban\\ -4+1.5\left(\frac{f}{925}-1\right) & ,\;metropolitan\;centers \end{array}\right.$$

Table 5 shows the log-distance representation of the COST 231-Walfisch–Ikegami model for NLOS. $L_{a}$ is an additional term given by $-0.08\left(h_{B}-h\right)d/\left[500{log}_{10}(d)\right]$, for $h_{B}<h$, and *d* < 500 m. This term generates a nonlinear trend when plotting path loss against distance on a logarithmic scale, affecting path loss only when the base station antenna is below building roofs. The model is applicable to the following ranges: 800≤f≤2000 MHz, 20≤d≤5000 m, $4\leq h_{B}\leq 50\;m$, and $1\leq h_{T}\leq 3\;m$.

The last model described in Table 5 is 3GPP [112]. It demonstrates validity in urban and suburban environments, covering the frequency range from 2 GHz to 6 GHz. The model applies to frequencies starting from 450 MHz and above when employed in rural environments. Considering the frequencies of interest of LoRa, Table 5 presents the results for rural environments. The parameter $d_{BP}$ is given by $2\pi h_{B}h_{T}f/300$. The NLOS model applies to both urban and suburban scenarios. The model is applicable for the following ranges: 10≤d≤10,000 m, $10\leq h_{B}\leq 150\;m,$ $1\leq h_{T}\leq 10\;m,$ 5≤h≤50 m, and 5≤W≤50 m.

### 2.2. Indoor Environments

Indoor propagation modelling typically involves predicting the propagation characteristics within a building. This environment experiences higher attenuation when compared with outdoor propagation, primarily due to the presence of walls and other obstacles. Most indoor models use a logarithmic decay of distance combined with losses induced by obstacles. The first method presented in Table 6 is the Motley–Keenan model [113], where $n_{F}$ is the number of floors in the propagation path, $\alpha_{F}$ is the floor attenuation in dB, $n_{W}$ is the number of walls in the propagation path, and $\alpha_{W}$ is the wall attenuation in dB. The attenuation with distance follows the free-space model, and the obstacles only affect the α parameter. The COST 231 multi-wall model follows a similar procedure [102]. Table 6 shows the representation for this model, where *L_C_* is a constant loss, $n_{Wi}$ is the number of walls of type *i*, and *b* is an empirical factor. The log-distance model with a path loss exponent different from free space is also employed for indoor environments [114]. Table 6 displays the representation of the Seidel–Rappaport model [92]. FAF is a function of the number of floors and building type, and *n* is the path loss exponent for the same floor.

The Indoor Dominant Path (IDP) model [115,116] is also present in Table 6, where $L_{W}$ is the wall loss, and $L_{B}$ is the interaction loss, which depends on the type of wall, the operating frequency, and the angle made by the propagation path. Tuan et al. [117] proposed a model with constants to account for some environment parameters. Table 6 shows the results of this model, where $G_{a}$ refers to the antenna gains, and $P_{1}$ and $P_{2}$ are associated with the angle of incidence to a wall, in the form ${(P}_{1},P_{2})=(\sin\left(\theta \right),1-\sin\left(\theta \right))$ or ${(P}_{1},P_{2})=\left({sin}^{2}\left(\theta \right),{\left[1-\sin\left(\theta \right)\right]}^{2}\right).$ The coefficients of $k_{1}$ to $k_{6}$ are to be determined based on measured data.

Barbosa et al. [118] established a parameterization for the path loss exponent based on the number of floors, yielding the results shown in Table 6. The parameters *a* and *b* are determined through a curve-fitting process based on measurements. Degli-Esposti et al. [119] proposed a model that includes a linear term as a function of distance, given by

(21) $$PL(d)=PL(d_{0})+10n{log}_{10}\left(\frac{d}{d_{0}}\right)+\gamma d$$

Other authors have also recommended employing a linear trend for indoor environments [102,120]. Table 6 shows the log-distance representation for this model. Due to the slower increase in ${log}_{10}\left(d\right)$ compared to *d*, the β component nearly exhibits linear growth with distance.

### 2.3. Within Vegetation Environments

Vegetation can significantly increase signal attenuation in communication systems. This observation has prompted the development of propagation models that incorporate environmental parameters. Several conventional empirical models calculate excess attenuation above free space in the form [121]

(22) $$L=Af^{B}d^{C}\;(dB)$$

The parameters *A*, *B*, and *C* were determined by curve-fitting to measured data. Table 7 presents the well-known models of this type. We converted the frequency from GHz to MHz for the models specified in GHz.

The models presented in Table 7 deviate from the conventional log-distance decay pattern. Given our aim to establish a uniform model throughout this work, it was necessary to carry out a study to examine the underlying principles that led to the formulation of the previous models. Weissberger [42] conducted a comprehensive study investigating the modelling of additional attenuation caused by propagation through vegetation. The conventional method for determining received power, expressed as $p_{r}=p_{t}g_{t}g_{r}e^{-ad}/l_{b0}$, assumes an exponential increase in loss with distance, where *a* represents the differential attenuation due to foliage, and $l_{b0}$ denotes the loss in the absence of vegetation. The path loss in dB is ${PL}_{b0}=L_{b0}-a_{1}d,$ with $a_{1}=10{log}_{10}\left(e\right)a$. However, Weissberger noted a discrepancy between this model and actual measurements, particularly for distances exceeding 15 m within vegetation. To address this deviation, the author introduced the Modified Exponential Loss (MED) model based on various sets of measurements.

We compared the outcomes obtained by applying the log-distance model with those derived from the model specified in Equation (22), using measurements presented in Weissberger’s article. Both models were determined by curve-fitting of measurements acquired for 910 MHz and 1.85 GHz, and each case involved computing the root mean square error (RMSE). The difference between root mean square errors ranged from 0.1 to 0.5 dB. This error indicates that both models exhibit comparable levels of accuracy. AI-Nuaimi and Hammoudeh [128] also proposed a model based on Equation (22) for the frequency of 11.2 GHz. We employed curve-fitting on the measurements of that study to determine the log-distance and MED models, and our findings led to the same conclusions. Therefore, the log-distance model could replace the MED models to predict the path loss within vegetation.

Goldman and Swenson proposed [129] the floating-intercept model in frequencies in the VHF and low UHF bands. They determined the model parameters via curve fitting. Fanimokun and Frolik [130] employed the close-in model for the frequency of 915 MHz, and Joshi et al. [131] applied the floating-intercept model to 300 MHz and 1900 MHz. All the presented models lack environmental parameters, which may lead to uncertain accuracy when applied to a different environment. Phaiboon and Somkurnpanich [132] do not include environments but developed models for different vegetation densities. The vegetation parameters employed in the log-distance models were the number of trees per square meter, leaf dimensions and the number of leaves per volume.

Table 8 shows models that include parameters from vegetation. The maximum attenuation model [133] uses two vegetation parameters. $A_{m}$ is the maximum attenuation, and ξ is the specific attenuation for a very short distance (dB/m).

The Azevedo–Santos trunk model is valid for environments where tree trunks dominate the propagation path [134]. $T_{D}$ is the tree density (trees/m^2^), and D is the average diameter of trunks in cm. The parameter $d_{m}$ is set to 60 m if *d* ≤ 60 or the maximum distance of prediction if *d* > 60. The model is valid for distances up to 600 m and for path loss values above those of free space. The log-distance representation of the Kurnaz–Helhel model [135] is shown in Table 8, where *k* is the trunk height gain. This parameter is given by $k=h_{ref}/h_{ex}$, where $h_{ref}$ represents the height of the tree part without leaves in a reference environment, and $h_{ex}$ is the height of the tree part without leaves in the examined environment. The Xiuming–Chunjiang model [136] was developed to estimate path loss in an apple orchard operating at 2.4 GHz. Table 8 describes the results, where *h* represents the antenna heights. Guo et al. [137] applied this model to an apple orchard, introducing the Leaf Area Index (LAI) parameter to determine the path loss exponent. The Azevedo–Santos foliage model [138] was designed to address the foliage zone, considering vegetation parameters such as foliage density, FD (%), tree density, $T_{D}\;(trees/m^{2})$, and the average tree canopy diameter, $D_{C}\;(m)$. Botella-Campos et al. [139] proposed a model detailed in Table 8, requiring parameters like path loss at a distance of one meter (*PL*(1 m)), the number of trees in the propagation path ($N_{t}$), and the average tree loss ($L_{v}$). Xiao et al. [140] introduced the BHF model, where $L_{0}$ represents an optimized offset value for path loss in dB, and ζ characterizes the coefficient for path loss caused by vegetation attenuation. The Phaiboon–Phokharatkul model [82] incorporates log-distance decay with an additional tree attenuation term. In Table 8, TAF denotes the tree attenuation factor.

**Table 8:** Parameters for models within vegetation.

Model	Component of *α*	Value (dB)	Component of *β*	Value (dB)
Maximum attenuation model [133]	$K_{\alpha }$	-	$K_{\beta }$	2
	$P_{1}\left(f\right)$	-	$F_{1}\left(f\right)$	-
	$P_{2}\left(A_{m},\xi \right)$	$A_{m}$	$F_{2}\left(A_{m},\xi \right)$	$-\frac{A_{m}e^{-\frac{\xi }{A_{m}}d}}{10{log}_{10}\left(d\right)},d>1$
Azevedo–Santos trunk model [134]	$K_{\alpha }$	-	$K_{\beta }$	2
	$P_{1}\left(f\right)$	$0.019f^{0.47}T_{D}D$	$F_{1}\left(f\right)$	$0.03f^{0.42}{\left(d_{m}-40\right)}^{-0.15}$
	$P_{2}\left(T_{D},D\right)$	$-0.026d_{m}T_{D}D$	$F_{2}\left(T_{D},D\right)$	$0.043{\left(d_{m}-40\right)}^{0.47}T_{D}D$ $-0.45{\left(d_{m}-40\right)}^{-0.15}$
Kurnaz–Helhel model [135]	$K_{\alpha }$	$-G_{B}{-G}_{T}+\left\{\begin{array}{cc} 0 & ,d\leq 200\\ -37.55 & d>200 \end{array}\right.$	$K_{\beta }$	$\left\{\begin{array}{cc} 2 & ,d\leq 200\\ 4 & ,d>200 \end{array}\right.$
	$P_{1}\left(f\right)$	-	$F_{1}\left(f\right)$	-
	$P_{2}\left(h_{B},h_{T}\right)$	${-20log}_{10}\left(h_{B}h_{T}\right)\;\;,d>200$	$F_{2}\left(h_{B},h_{T}\right)$	-
	$P_{3}\left(k\right)$	-	$F_{3}\left(k\right)$	$k\frac{258.4d-3857}{(d+11,900){log}_{10}\left(d\right)},\;d>1$
Xiuming–Chunjiang model [136]	$K_{\alpha }$	4.2	$K_{\beta }$	2.273
	$P_{1}\left(f\right)$	-	$F_{1}\left(f\right)$	-
	$P_{2}\left(h\right)$	−1.133h	$F_{2}\left(h\right)$	0.0507h
Azevedo–Santos foliage model [138]	$K_{\alpha }$	0.61	$K_{\beta }$	2.27
	$P_{1}\left(f\right)$	$-4.65e^{37.1\left(\frac{1}{2400^{0.5}}-\frac{1}{f^{0.5}}\right)}$	$F_{1}\left(f\right)$	-
	$P_{2}\left(FD\right)$	$\left[0.31e^{37.1\left(\frac{1}{2400^{0.5}}-\frac{1}{f^{0.5}}\right)}-0.1\right]FD$	$F_{2}\left(FD\right)$	-
	$P_{3}\left(T_{D},D_{C}\right)$	$\left[7.68\times 10^{-9}f^{2}-4.26\times 10^{-3}f-19.85\right]T_{D}D_{C}$	$F_{3}\left(T_{D},D_{C}\right)$	$\left[-1.1\times 10^{-9}f^{2}+6.1\times 10^{-4}f+2.84\right]T_{D}D_{C}$
Botella-Campos et al. model [139]	$K_{\alpha }$	$PL\left(1m\right)-27.56$	$K_{\beta }$	2
	$P_{1}\left(f\right)$	${-20log}_{10}\left(f\right)$	$F_{1}\left(f\right)$	-
	$P_{2}\left(N_{t},L_{v}\right)$	$N_{t}L_{v}$	$F_{2}\left(N_{t},L_{v}\right)$	-
BHF model [140]	$K_{\alpha }$	$L_{0}-27.56$	$K_{\beta }$	n
	$P_{1}\left(f\right)$	-	$F_{1}\left(f\right)$	-
	$P_{2}\left(\zeta \right)$	-	$F_{2}\left(\zeta \right)$	$\zeta \frac{\tanh\left(\frac{d}{20}\right)}{10{log}_{10}\left(d\right)},\;d>1$
Phaiboon–Phokharatkul model [82]	$K_{\alpha }$	-	$K_{\beta }$	n
	$P_{1}\left(f\right)$	-	$F_{1}\left(f\right)$	-
	$P_{2}\left(TAF\right)$	$\sum_{i=1}^{M}{TAF}_{i}$	$F_{2}\left(TAF\right)$	-

## 3. Active Measurement Propagation Models

The standard approach of prior models predicts network behaviour in a specific environment using analytical expectations or data from similar settings. In contrast, posterior models acknowledge the inherent limitations of such assumptions and necessitate environmental measurements for accurate predictions. These latter models essentially define a measurement and prediction methodology that can be used at new locations [13] and address the lack of robustness, insufficient efficiency, and ineffectiveness of conventional models in challenging environments [141].

More specifically, an active measurement propagation model utilizes empirical data collected from field measurements to characterize the radio wave propagation environment. These models can incorporate parameters such as path loss, shadowing, fading, and multipath effects, offering a comprehensive framework for assessing signal propagation in diverse scenarios.

One approach involves curve fitting of existing empirical models. This traditional methodology frequently uses empirical models and tunes its parameters with real-world measurements gathered across the deployment area, customizing it to the specific environment. A common methodology is to add the root mean square error as a constant term to minimize prediction errors. Conversely, Udofia et al. [142] proposed a more innovative methodology by using a composite function of empirical model residuals rather than directly adding RMSE to the model. The composite function was derived by fitting a trend line to the graph of prediction residuals against the Okumura–Hata model’s path loss predictions, leading to a correction term added to the Okumura–Hata path loss model for urban areas, estimated to be $\left[0.1457466236\hat{Y}\left(d\right)-0.641993705\hat{Y}\left(d\right)\right]/[\hat{Y}\left(d\right)-99.3737369]$, where $\hat{Y}\left(d\right)$ is the Okumura–Hata predicted path loss. When comparing with the measured path loss, the un-tuned Okumura–Hata predictions had an RMSE of 16 dB, with an accuracy of 89%, while the tuned model metrics were 2 dB and 99%, respectively. However, by using a simple RMSE-based constant, the metrics were 5 dB and 97%, respectively, highlighting the relevance of adding a correcting term that considers the environmental characteristics.

The interpretability of curve-fitting models is a notable advantage, as it provides insights into how various factors affect signal strength, thus facilitating a clear understanding of propagation behaviours. However, this approach may encounter limitations in accurately capturing the complexities of real-world propagation, particularly in heterogeneous environments. Machine learning was developed to address these types of limitations by allowing models to learn the patterns directly from data. The models can further be used to model multiple parameters of the wireless communication channel, as discussed by Aldossari and Chen [11]. Nevertheless, they are particularly interesting for path loss analysis [141] since measurement-based models can be constructed using machine learning techniques.

These machine learning models can discern intricate relationships between multiple factors and predict signal strength for new locations based on learned patterns from data. Therefore, these methods can be used as an alternative to empirical and deterministic models, framing path loss prediction as a supervised regression problem. Moraitis et al. [143] reported that these models can substantially outperform empirical models in the application site, where the RMSE ranged from 4 to 7 dB in the examined rural areas. Their approach employed principal component analysis to examine the correlation between the examined features and the measured path loss. It was concluded that the transmitting and receiving antenna’s heights, path visibility, and the direct distance between the antennas are the more relevant characteristics for the models. The best performance was attained when using an Artificial Neural Network (ANN).

Machine learning models are particularly useful in developing models for environments where an empirical method could not be directly applied. Gomes et al. [144] propose using k-nearest neighbours with knowledge-based theory to develop a model suitable to non-homogeneous paths and climates. Specifically, the measured points considered attributes related to the terrain morphology collected locally and from satellite aerial optical images. The authors reported a low error in the forecasts. Liu et al. [65] introduced an alternative method to estimate path loss for long-range LoRa communication in challenging environments. This approach utilizes remote sensing data, specifically multi-spectral images, to automatically identify various types of land cover along the signal path. The identified land cover information is then intake into a bidirectional Long Short-Term Memory Neural Network (LSTM) followed by a convolutional layer. The effectiveness of this model is validated using real-world LoRa data, demonstrating a notable reduction in path loss estimation error to below 4 dB. The employment of deep learning techniques is particularly significant for path loss estimation, as these models can directly learn relevant patterns from the data without requiring complex feature engineering methods, which typically involve laborious feature creation and selection processes.

Machine learning-based models, particularly ANN, have found widespread success in path loss analysis across various environments [145,146,147,148,149,150,151,152,153,154,155,156,157,158,159,160]. They hold promise for achieving higher accuracy, particularly in settings with intricate propagation characteristics, as underscored by Hakim et al. [84]. However, their black-box nature may pose challenges in interpreting and explaining model predictions, especially when employing deep learning methods. Furthermore, the requirement for extensive and diverse datasets to train the models may be impractical in certain environments. Additionally, it is crucial to observe if the models can achieve generalization for new locations, as reported by Rofi et al. [66], who noted that their proposed method performed optimally when tested on the same dataset.

Hybrid approaches can combine machine learning techniques with traditional propagation models or curve-fitting methods to leverage the strengths of both approaches. Two main approaches were identified in this domain. The first deals with correcting the empirical model’s estimations, while the second consists of aiding in selecting the most suitable empirical model based on the classification of the terrain.

Thrane et al. [161] introduced a methodology for the first approach, employing a model based on a convolutional neural network for satellite image analysis. This machine learning model was used to learn how to correct the estimated path loss produced by a standard path loss model. The evaluation revealed that the model can improve path loss predictions for unseen locations, showing an improvement of approximately 1 dB for 811 MHz and 4.7 dB for 2630 MHz.

Demetri et al. [45] introduced a method for the second approach. They utilized pixel-based Support Vector Machines (SVMs) to analyze multispectral images acquired through remote sensing. The automated classification of environmental types along communication links facilitates the interpretation of observed signal attenuation. By employing SVM with radial basis function kernels and the one-against-all strategy, they achieved an accuracy exceeding 90%. Subsequently, this model enables automated landscape analysis per link, leveraging the Okumura–Hata model to estimate expected received power. Validation with over 8000 real-world samples demonstrated the automated approach’s capability to predict signal power within an error margin of approximately 10 dBm, affirming its efficacy in accurately characterizing signal propagation across diverse environments.

From the examined literature, it is noticeable that active measurement-based propagation models offer substantial advantages for LoRa network planning and optimization. Firstly, they leverage real-world data collected through active measurements, leading to more accurate LoRa signal propagation predictions than empirical models. This enhanced accuracy provides a more reliable foundation for network design and optimization efforts. Secondly, these models exhibit adaptability across varied environments and operational contexts. By incorporating real-world data from the specific deployment scenario, they can produce customized propagation predictions that account for local factors. However, verifying the models’ generalization capabilities remains imperative. Finally, active measurements play a crucial role in validating and refining the models. By comparing model predictions with empirical on-site measurements, researchers can ensure the model’s effectiveness across different deployment scenarios, fostering continuous improvement.

## 4. Propagation Measurements

Most empirical propagation models use a log-distance formulation. Equation (1) defines the path loss at a reference distance of $d_{0}$, typically corresponding to the free space loss. Several researchers adopt $d_{0}=1\;m$, while others opt for values such as 100 m or 1 km [162]. For example, the SUI model employs $d_{0}=100\;m$, while the Lee model sets $d_{0}=1\;km$. However, these models incorporate additional losses, resulting in a path loss ${PL(d}_{0})$ that may diverge from that of free space. For a better understanding, we utilized measurements from published works. Moreover, our analysis extends to the observation that some models deviate from a log-distance trend, as revealed in prior studies. Herein, we delve into the examination of the actual tendencies extracted from measurement data.

Many authors choose to present measurement results through graphical representations. We extracted pertinent information from these graphs to calculate the path loss. Subsequently, we applied curve-fitting techniques to infer the parameters of Equation (2), specifically α and β. The parameter $d_{0}$ was determined via Equation (4), representing the distance at which the path loss intersects that of free space. Additionally, we determined the parameter *n* of Equation (1) for $PL\left(d_{0}\right)={PL}_{FS}\left(1\;m\right).$ Table 9 presents the outcomes of this analysis with measurements acquired in outdoor environments. The results do not include LoRa measurements because we will describe this case later. The root mean square errors for the difference between measurements and models are denoted as RMSE and RMSE_1_ for Equation (2) and Equation (1), respectively.

**Figure 1:**
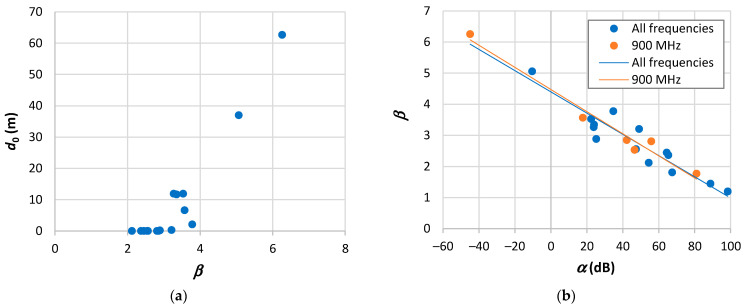
Results derived from measurement data: (**a**) relationship between the reference distance and *β* parameters; (**b**) relationship between *β* and *α*.

Comparing β with *n* in Table 9, we may observe that the highest differences between both occur when β falls below the path loss exponent of free space [161,167,176] and for the highest β values [165,168]. Examining $d_{0}$, we find ten values below or around 1 m, four around 10 m, and two ranging from 30 to 60 m. As illustrated in Figure 1a, there appears to be a trend of $d_{0}$ to increase with β. Figure 1b presents the parameter β plotted as a function of α. This outcome reveals a linear trend, indicating a decline in β as α increases and corroborating the observation of $d_{0}$ increasing with β. This tendency is independent of frequency, as demonstrated in the case of the 900 MHz band. The high values of RMSE observed in Table 9 are typically associated with a substantial spread of path loss at adjacent points. From the $d_{0}$ results shown in Table 9, there is no basis for the assigned values of 100 m and 1 km.

**Figure 2:**
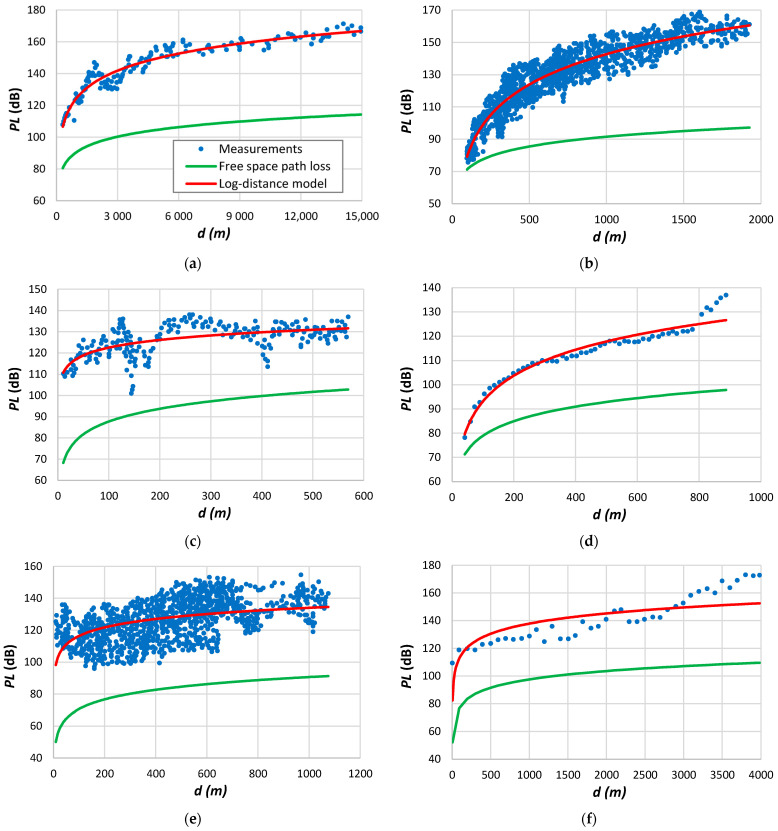
Measurement data: (**a**) Walfisch et al. [111] (α=17.80, β=3.57); (**b**) Phillips et al. [165] (α=−44.96, β=6.26); (**c**) Meza et al. [176] (α=98.41, β=1.20); (**d**) Ibhaze et al. [172] (α=22.4, β=3.53); (**e**) Thrane et al. [161] (α=81.01, β=1.77); (**f**) Waheed et al. [170] (α=64.48, β=2.45).

Some effects may not be immediately evident when comparing the results in Table 9. Figure 2 displays specific cases of the data used to construct Table 9. The examples aim to illustrate various situations related to the measurements found in the literature.

Figure 2a shows the measurement data acquired in Philadelphia, corresponding to the fifth row of results in Table 9. Averaging the path loss measurements removed small-scale fading. We may verify a good agreement with the log-distance model, as found in several cases of measurement data. Figure 2b shows measurements taken from the COST-231 dataset [165]. These results also follow a log-distance tendency, giving the highest values of β and $d_{0}$ in Table 9. The measurement results in Figure 2c reveal significant variability around log-distance decay. The data collection employed a van along a designated route within the urban area of Tanguá, Brazil. The parameter β determined by the log-distance model is 1.2, considerably lower than the free-space loss. The signal fluctuation implies varying obstacle densities along the propagation path between the transmitter and the receiver. Figure 2d illustrates a scenario where the path loss experiences a notable increase during the final segment of the measurements. This divergence signifies the inapplicability of a uniform model to all data. By curve-fitting data just to distances up to 800 m, the root mean square error decreases from 3.02 dB to 1.1 dB, while $d_{0}$ diminishes from 11.9 m to 5 m. The results presented in Figure 2e depict data obtained from a vast area, resulting in a remarkable root mean square error related to the log-distance decay. Consequently, the challenge arises in developing a model that accurately tracks the data, even for the data locations. The data depicted in Figure 2f were acquired using a mobile phone with a transmitter positioned at a height of 70 m. Notably, measurements taken at the closest distance from the transmitter exhibit remarkably high values, resulting in a substantially low reference distance, as indicated in the eleventh entry of Table 9. One plausible explanation for this high attenuation is the impact of the radiation pattern, particularly in positions near the transmitter. Figure 2f reveals distinct zones displaying varied path loss tendencies, suggesting the employment of different propagation models for fitting the data accurately.

The preceding methodology was applied to indoor environments, yielding the findings presented in Table 10. These results reveal that the reference distance $d_{0}$ typically falls within or below the range of one meter for indoor environments. Additionally, there is an observable trend of decreasing β as α increases, albeit with a more dispersed alignment when compared to outdoor outcomes. It is worth highlighting the elevated values acquired when the propagation path contains multiple floors.

**Figure 3:**
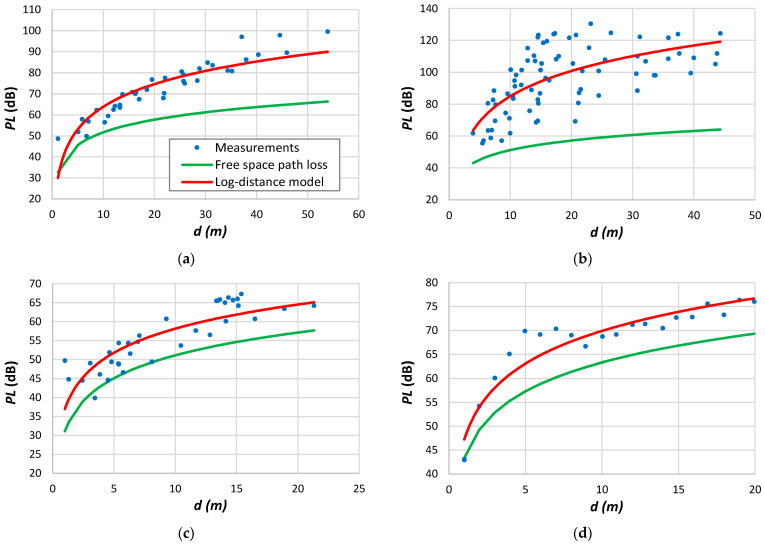
Measurement data: (**a**) Seidel et al. [92] (α=28.01, β=3.58); (**b**) COST 231 [102] (α=32.04, β=5.29); (**c**) Degli-Esposti et al. [177] (α=36.97, β=2.12); (**d**) Al-Samman et al. [179] (α=47.23, β=2.27).

Figure 3 illustrates several scenarios of measured data within indoor environments. In Figure 3a, measurements were taken on the same floor, whereas in Figure 3b, the measurements refer to data collected across four floors. The latter case demonstrates significant fluctuations around the average, resulting in a high root mean square error, as evidenced in the fourth line of Table 10. While a log-distance model can be employed, incorporating losses attributed to walls and floors increases accuracy, as emphasized by Degli-Esposti et al. [177]. Figure 3c depicts measured outcomes obtained in an office setting, while Figure 3d shows results collected in a corridor.

**Figure 4:**
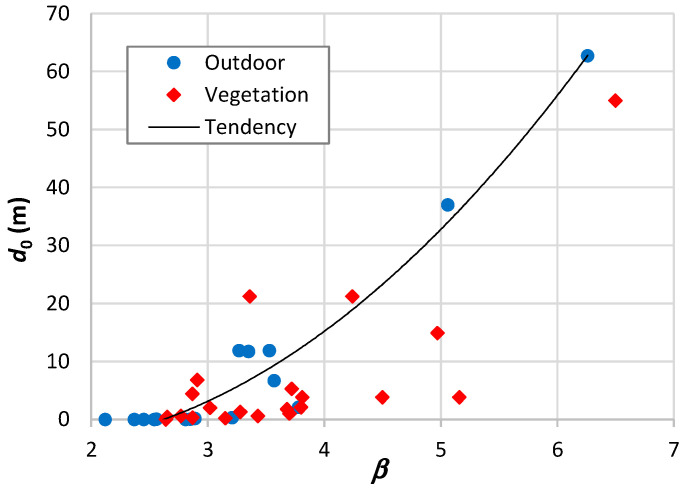
Reference distance as a function of *β*.

Various authors have conducted measurements on propagation within vegetation, providing the data presented in Table 11. The propagation range within vegetation is notably shorter compared to outdoor environments. Another observation is that the reference distance is around or below one meter in most cases. In such situations, the close-in model proves to be effective, yielding a root mean square error increase below 0.6 dB. Similar to outdoor environments, there is a tendency for $d_{0}$ to increase with β, as illustrated in Figure 4. From the results of this figure, it is noteworthy that some values deviate substantially from this trend. Through representative measurement examples, we will elucidate a reason for this divergence.

**Figure 5:**
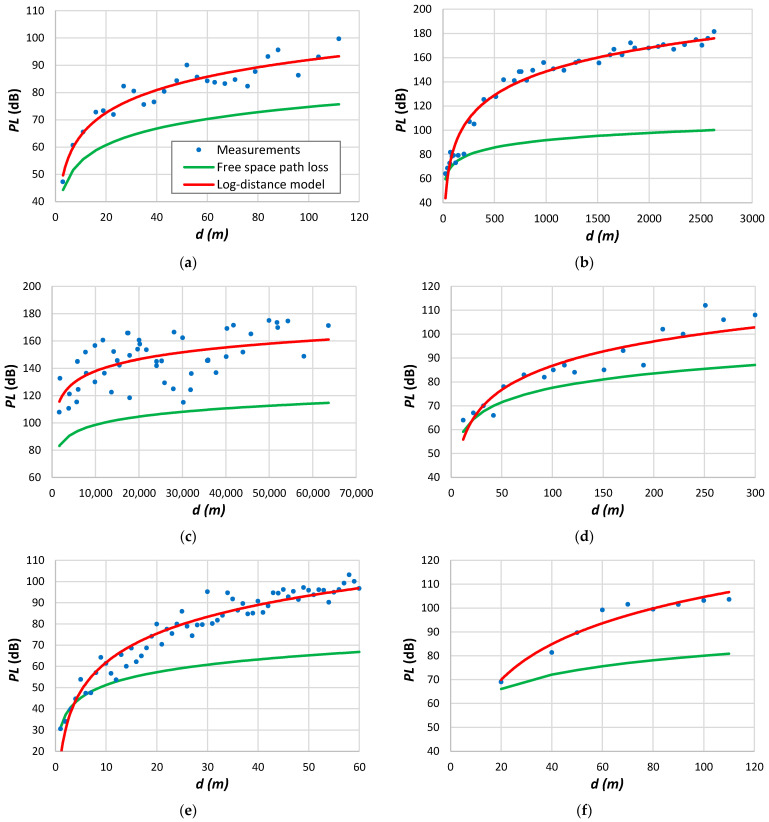
Measurement data: (**a**) Rogers et al. [184] (α=36.36, β=2.77); (**b**) Hejselbæk et al. [183] (α=−46.62, β=6.50); (**c**) Jawhly et al. [182] (α=23.26, β=2.87); (**d**) Phaiboon et al. [132] (α=19.53, β=3.36); (**e**) Azevedo et al. [138] (α=16.87, β=4.50); (**f**) Azevedo et al. [189] (α=5.17, β=4.97).

Figure 5 presents measurement results that serve to clarify the previous issues. Figure 5a represents the measurement data employed to derive the outcomes presented in the tenth row of Table 11. This outcome is a typical example of data following the log-distance model with a reference distance of around one meter. Figure 5b displays measurements leading to the highest value of $d_{0}.$ Figure 5c illustrates a scenario characterized by significant variability in data points around the trend line, emphasizing a range exceeding 60 km. This long-range highlights the need to establish distinct models for regions exhibiting an approximately homogeneous environment.

Let us consider the cases diverging from the expected tendency of $d_{0}$. An illustrative instance of this phenomenon is evident in the outcomes of the twelfth row of Table 11, characterized by parameters β=3.36 and $d_{0}=21.2\;m$. The value of $d_{0}$ is high for a relatively low β. Figure 5d shows a discernible lower attenuation below 200 m compared to values beyond this threshold. Employing the log-distance model with dual slopes yields β=2.41 for distances below 200 m and β=3.17 for distances above 200 m. The reference distance diminishes to approximately 4 m, aligning with the observed trend in Figure 4.

A different situation occurs for the results in rows six and seventeen of Table 11, with the values of $d_{0}$ much lower than the trend. Figure 5e shows the case for 870 MHz. The data appears to follow the log-distance model for all ranges. When comparing the results to those of Figure 5b, the primary distinction lies in the range of measurements. Figure 5b exhibits a range of 2600 m, whereas Figure 5e shows a significantly reduced range of 60 m. This implies that the reference distance cannot be substantial when the range is limited. Similar conclusions also occur for indoor environments with high attenuation in short ranges. Figure 5f refers to the results presented in the twentieth row of Table 11. In this case, the reference is lower than expected but exceeds that of Figure 5e because of the extended range.

## 5. Propagation in LoRa Systems

The study focusing on LoRa primarily operates within the Industrial, Scientific, and Medical (ISM) band of 900 MHz, with 868 MHz employed in Europe and 915 MHz in North America.

### 5.1. RSSI, SNR and ESP

The extended range achieved in LoRa systems is due to their operation with Signal-to-Noise Ratio (SNR) values below 0 dB. For example, a typical Semtech LoRa radio operates with a minimum SNR value of −20 dB [191].

The study outlined in the Introduction regarding LoRa literature reveals that numerous authors utilize the term Received Signal Strength Indicator (RSSI) to denote the received signal power. RSSI serves as a measure of the received power given by the receiving radio. However, this parameter contains both the power of the received signal and any accompanying noise and interference [192]. The RSSI values much lower than 0 dB essentially mirrors the power of the noise and interference. Consequently, determining the path loss from RSSI in such scenarios becomes impractical. To mitigate this limitation, the authors typically sum the SNR to the RSSI to approximate the received signal power for SNR < 0 dB and consider the RSSI when SNR ≥ 0 dB.

Some authors advocate for utilizing Effective Signal Power (ESP) over RSS to define the received signal power [19,193,194,195]. The ESP is derived from the signal-to-noise ratio and is given by

(23) $$ESP\left(dBm\right)=RSS\left(dBm\right)+SNR\left(dB\right)-10\left(1+10^{0.1SNR(dB)}\right)$$

The path loss is then defined by

(24) $$PL\left(dB\right)=P_{T}(dBm)+G_{T}(dBi)+G_{R}(dBi)-ESP(dBm)$$

where $P_{T}$ is the transmitted power, $G_{T}$ is the transmitter antenna gain, and $G_{R}$ is the receiver antenna gain.

### 5.2. RSSI Calibration

The RSSI is a metric given by LoRa radios to indicate the received power. Gutiérrez-Gómez et al. [80] underscored the imperative need for RSSI calibration. In their study, Benaissa et al. [196] noted a shift of 6 dB disparity between the RSSI reported by a LoRa radio operating at 868 MHz and the power measured by a spectrum analyzer. Linka et al. [58] evaluated the LoRa SX1272 chipset’s RSSI accuracy. Their findings revealed that the radio consistently measured values lower than those indicated by the spectrum analyzer. Specifically, they reported a discrepancy of approximately −21 dB for a received power of −30 dBm and roughly −12 dB for a signal strength of −80 dBm. Some studies revealed a correlation between the RSSI and the Spreading Factor (SF) of LoRa [24,197]. A higher SF yields a more elevated processing gain, thereby augmenting the range. However, as the path loss should remain unaffected by the measurement system, it mandates a calibration procedure.

**Figure 6:**
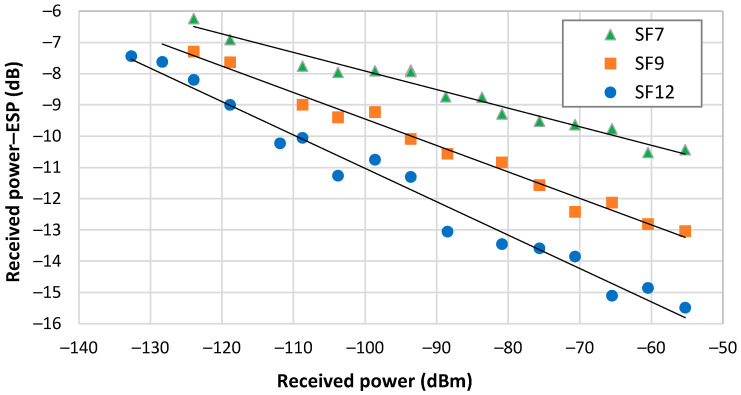
ESP accuracy verification.

To test the accuracy of LoRa radio RSSI readings, we devised an experimental setup following the calibration procedure outlined by Ameloot et al. [198]. Utilizing the signal generator SMC100A from Rohde and Schwarz [199] with an output power of 0 dBm as the transmitter, we measured the received power using a spectrum analyzer FSH8, also from Rohde and Schwarz [200]. Stepped attenuators positioned between the transmitter and the receiver, connected via coaxial cables, regulated the attenuation introduced in the system. We evaluated the RFM95 Adafruit LoRa modules operating at 868 MHz, housing the SX1276 transceivers from Semtech [201], with an output power of 0 dBm and a frequency bandwidth of 125 kHz. We measured the RSSI and SNR for three SF values, 7, 9, and 12. The corresponding receiver sensitivity was −123 dBm, −129 dBm, and −136 dBm, respectively. Afterwards, we applied Equation (23) to calculate the ESP and subsequently compared these results with the received power provided by the spectrum analyzer. Figure 6 shows the outcomes. Observing this figure, it is evident that the ESP values are consistently lower than the received power reported by the spectrum analyzer. In addition, the difference in results is not uniform across the range of the received power, aligning with findings in [58]. This variation exhibits an almost linear trend, which facilitates that calibration procedure. Furthermore, it is notable that the difference between received power and ESP widens with increasing spreading factors.

### 5.3. Path Loss Measurements with LoRa Radios

Following the methodology previously employed for conventional communication systems, we extracted the log-distance parameters from the curve fitting of LoRa measurements. When analyzing the measurement results, we encountered a notable deviation from the results outlined in Section 4. In numerous instances, our analysis of LoRa measurements unveiled a phenomenon where the average attenuation tends to stabilize. This behaviour typically occurs around the receiver’s sensitivity threshold. This situation can be justified by the extended transitional region of LoRa systems, resulting in non-uniform coverage [45]. Such behaviour has the potential to skew the path loss parameters.

**Figure 7:**
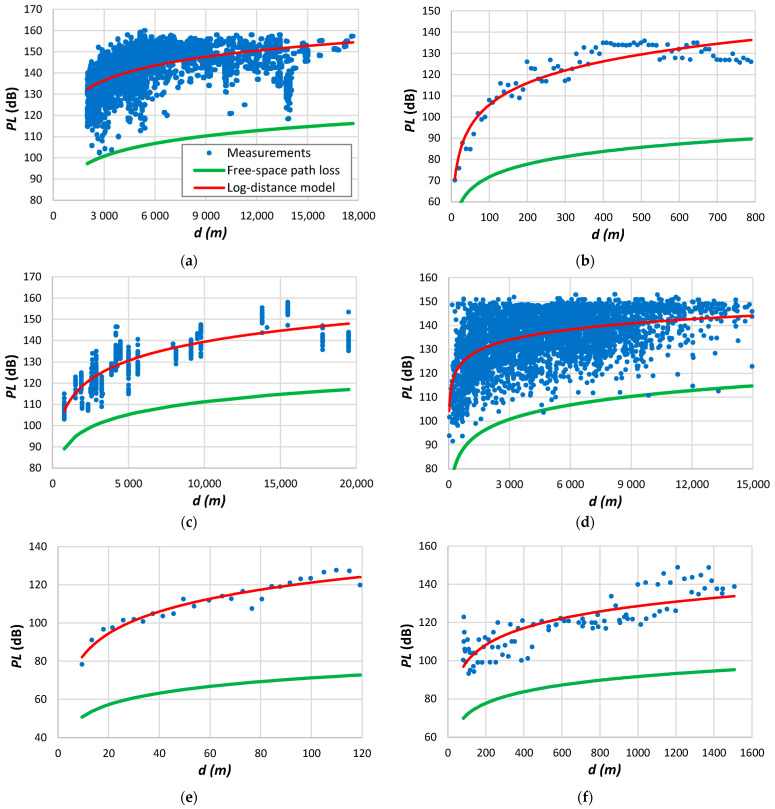
Measurement data: (**a**) Petäjäjärvi et al. [17]; (**b**) Avila-Campos et al. [22]; (**c**) Chall et al. [32]; (**d**) Stusek et al. [33]; (**e**) Muppala et al. [205]; (**f**) Batalha et al. [28].

To address this issue, we followed two distinct approaches to derive the log-distance parameters. Firstly, we utilized all accessible data to extract the desired parameters. Secondly, we focused on data up to the transitional zone. This zone can be defined as either the point where the system starts encountering packet loss or a distance where the ESP exceeds the receiver’s sensitivity by roughly one standard deviation of the data. Table 12 presents the obtained results. As confirmed by the results of this table, the transitional region manifests in data collected within distances spanning several kilometres. Figure 7 illustrates measurement examples to aid in comprehending some findings presented in Table 12.

**Table 12:** Parameters derived from measurements with LoRa.

Reference	Environment	Range (m)	*f* (MHz)	All Data				Below Transitional Region			
				*α* (dB)	*β*	RMSE	$d_{0}(m)$	*α* (dB)	*β*	RMSE	$d_{0}(m)$
[86]	Forest	5–100	433	7.18	5.97	6.31	2.8	-	-	-	-
[88]	Palm tree	10–90	433	−16.59	6.56	2.24	8.2	-	-	-	-
[202]	Suburban	250–8150	868	12.00	3.48	3.36	19.9	5.17	3.72	3.86	32.9
[17]	Urban	2000–18,000	868	55.18	2.34	8.29	10^−7^	−10.38	4.19	8.05	80.0
[203]	Rural	50–900	868	30.62	3.39	2.29	1.1	-	-	-	-
[19]	Suburban	100–20,000	868	93.75	1.47	5.71	-	72.99	2.13	5.56	-
[204]	Urban	80–6000	868	54.88	2.45	7.22	5 × 10^−6^	49.46	2.63	7.4	0.001
[20]	Urban	30–6000	868	57.24	2.41	8.62	5 × 10^−7^	89.52	1.28	6.80	-
[21]	Suburban	5–50	915	38.00	3.05	3.63	0.3	-	-	-	-
[45]	Suburban	900–50,000	868	39.49	2.18	3.83	2 × 10^−5^	43.51	2.05	4.02	-
[46]	Urban	60–2300	868	33.18	3.24	6.76	0.69	33.74	3.20	6.96	0.62
[22]	Forest	10–800	915	37.54	3.41	5.39	0.38	-	-	-	-
[15]	Campus	10–210	915	41.23	2.73	3.21	0.05	-	-	-	-
[23]	Rural	1–1000	868	91.79	1.87	9.96	-	83.27	2.19	10.54	-
[32]	Campus	5–155	868	42.14	3.41	10.41	0.17	-	-	-	-
[32]	Rural	700–20,000	868	21.86	2.94	6.78	9.9	22.14	2.93	6.31	9.5
[33]	Urban	1–15,000	868	83.48	1.45	8.81	-	59.83	2.33	12.50	3 × 10^−9^
[34]	Campus	50–1000	868	−4.81	5.30	13.71	12.3	−37.04	6.76	16.27	27.1
[34]	Forest	40–900	868	46.04	3.63	8.85	0.12	23.18	4.68	11.38	2.0
[34]	Urban	1–1000	868	100.9	1.82	7.00	-	63.29	4.22	7.47	0.04
[35]	Urban	5–1000	868	72.39	1.85	4.88	-	59.14	2.57	4.07	10^−5^
[65]	Campus	50–3500	868	70.93	2.25	7.61	-	57.21	2.81	10.85	6 × 10^−4^
[205]	Indoor	10–120	868	44.81	3.82	3.32	0.18	-	-	-	-
[81]	Forest	400–2000	920	44.45	2.53	8.16	0.004	-	-	-	-
[89]	Forest	150–1000	920	33.27	2.94	9.11	0.68	-	-	-	-
[6]	Forest	60–2500	915	50.10	2.78	8.99	0.004	−3.27	4.75	6.63	18.5
[28]	Suburban	80–1600	915	34.21	3.12	4.23	0.59	-	-	-	-
[28]	Suburban	80–1600	915	24.42	3.59	7.15	3.1	-	-	-	-
[28]	Suburban	800–2800	915	9.07	4.16	7.28	11.2	25.28	3.56	5.50	2.6
[28]	Suburban	800–2800	915	17.42	3.89	6.82	5.7	18.62	3.85	6.86	5.1
[28]	Suburban	500–2600	915	−0.76	4.62	8.10	17.4	23.38	3.69	6.62	3.1
[28]	Suburban	500–1900	915	4.50	4.67	10.49	10.4	28.11	3.60	7.41	1.7

Figure 7a shows measurements conducted by Petäjäjärvi et al. [17] in Oulu, Finland, utilizing an end device attached to a vehicle. The data reveal significant variability around the average, attributed to diverse obstruction densities between the transmitter and the receiver. The transitional region emerges above 6 km. According to the findings from Table 12, in this scenario, the parameter $d_{0}$ is 10^−6^ m when utilizing all available data to derive the log-distance parameters. However, this value rises to 80 m when restricting the analysis to distances up to 6 km. Remarkably, the authors noted a significant packet loss ratio of 74% between 10 and 15 km, with only a few packets received beyond the 15 km threshold.

Figure 7b depicts the data collected in a forest environment. Once more, it is evident that a single-slope model cannot accurately represent the data. Chall et al. [32] collected the data depicted in Figure 7c from a rural environment. These measurements depict the path loss variation at each position, facilitating the extraction of the small-scale fading effect. The results presented in Table 12 for this example indicate a higher $d_{0}$ value compared to the trend outlined in Figure 4. However, the authors only gathered data above 700 m and across a wide range of distances. We observed this effect in other similar situations. The results presented in Figure 7d exhibit significant deviation from the average, spanning from the free-space path loss to the limit imposed by the receiver sensitivity. Figure 7e illustrates measurements acquired in an indoor environment [205]. The authors also determined the variation of the received signal per position, obtaining a small-scale fading up to approximately 10 dB. Batalha et al. [28] conducted measurements on various routes in Belém, Brazil (Figure 7f). Focusing on data from a single route provides better insights into various obstruction tendencies. With the gateway positioned on the roof of a 50 m building and the end device mounted on a vehicle, significant attenuation is noticeable near the transmitter due to antenna gain loss and potential diffraction from the building roof.

### 5.4. Evaluation of Propagation Models

The objective of this section is to compare model outcomes with measurement data. The study focused on outdoor environments due to the higher number of developed empirical propagation models and the large number of measurement results. In most cases, the authors collected LoRa measurements with terminal antenna heights ranging between 2 and 3 m. Therefore, we evaluated results for 870 MHz and a terminal antenna height of 3 m. Altering the antenna height to 2 m resulted in an average attenuation decrease of 2.3 dB, with most models showing differences below 2.5 dB.

Given the fixed frequency, we undertook a comparison of the results of $P_{1}\left(f\right)$ from Equation (5). As this parameter encapsulates frequency attenuation beyond free-space loss, we anticipated some consistency in model outcomes. However, the findings reveal significant variability, with values spanning from 0 to 45 dB and one model indicating a value close to −40 dB.

**Figure 8:**
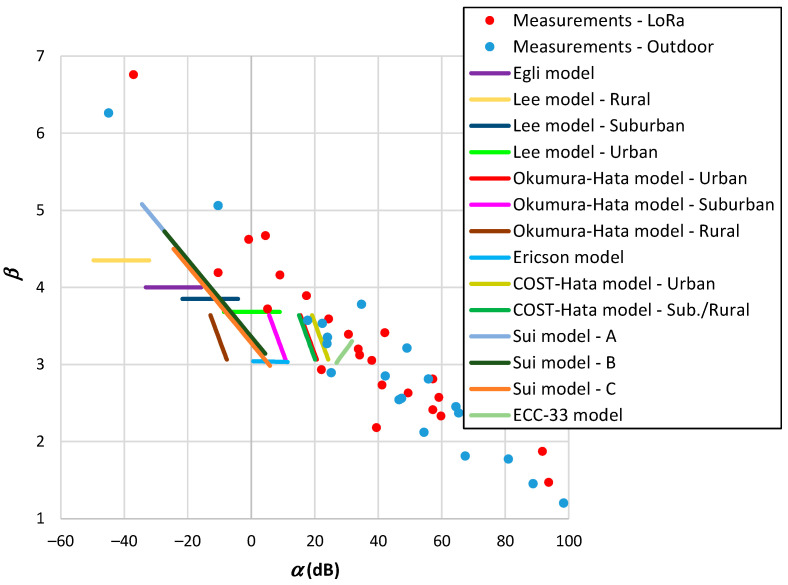
Parameter *β* as a function of *α* for the models described in Table 1, Table 3, and Table 4.

The outdoor models of Table 1, Table 3, and Table 4 employ distance, frequency, and antenna heights as input parameters. We fixed the frequency and antenna height of the terminal device ($h_{T}$) and varied the gateway antenna height ($h_{B}$) within the range from 20 to 150 m. Figure 8 displays the results showing the relationship between α and β. From this figure, it is evident the propagation models do not encompass the range of β values observed in the measurement results. Some models assume β to be constant and limited variations in α. Since many of these models were developed based on the Okumura–Hata framework, they exhibit similar trends. Notably, the SUI model demonstrates a broader range of variation in β. The model results suggest an average effect of the measurements carried out over a wide range of values. Another observation is that the α parameters provided by the models are lower than those obtained from measurements. This trend suggests a higher reference distance ($d_{0}$) than that derived from measurements. It is worth mentioning that several authors consider this reference distance to be 100 m or even 1 km. Therefore, the accuracy of the models in estimating the received signal of a LoRa system is low.

**Figure 9:**
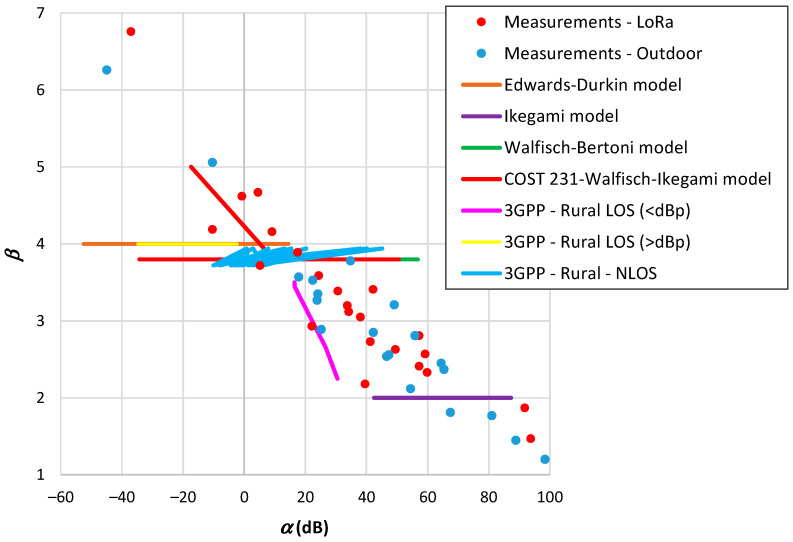
Parameter *β* as a function of *α* for the models described in Table 5.

The outdoor models outlined in Table 5 incorporate additional input parameters. The results depicted in Figure 9 were determined similarly to those in Figure 8, with discrete variations in model parameters. The representation of parameter β as a function of α reveals similarities to those observed in Figure 8, such as several cases with a constant β. The COST231-Walfish–Ikegami model exhibits a broader range of variation in β, potentially due to its foundation on other models. Nevertheless, this model does not cover the range of β<3.8. The 3GPP model for Rural LOS demonstrates a larger variation in β, but solely for distances falling below the breakpoint.

**Figure 10:**
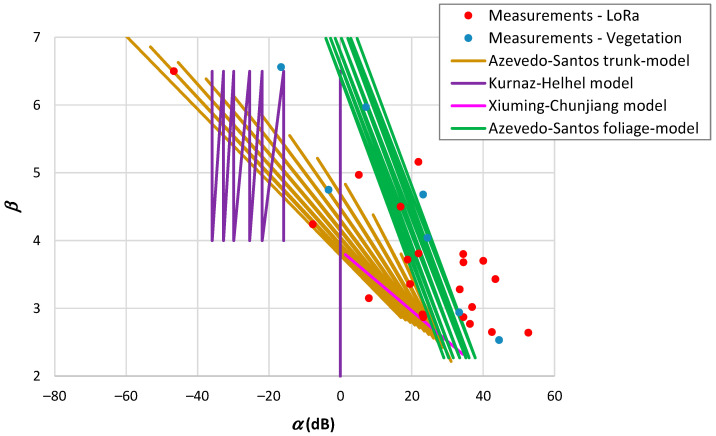
Parameter *β* as a function of *α* for the models described in Table 8.

We have also compared the outcomes of propagation models developed for vegetation environments with actual measurement data. Upon examination of Table 8, it becomes clear that some models are semi-empirical, requiring the measurement of propagation parameters within the environment, while other models extract directly these parameters from the environment. Figure 10 depicts the correlation between α and β for the second category of models.

The propagation models for vegetation depicted in Figure 10 exhibit a broader range of β values. The Kurnaz–Helhel model, designed for pine forests, potentially indicates a departure of α from the observed measurement results. Conversely, the Xiuming–Chunjiang model, devised for an apple orchard, demonstrates a narrower range in β. The Azevedo–Santos trunk and the Azevedo–Santos foliage models overlap more closely with the measured outcomes.

### 5.5. Multi-Slope Log-Distance Model

One problem with published models based on logarithmic distance is that authors often apply the same model to all data. This form does not consider the non-homogeneity of the propagation environment. Figure 7a,d demonstrate the limited accuracy of employing a single-slope model.

Several works have employed a dual-slope model [23,27,34,134,206,207,208,209], with some considering the free-space path loss for the first region. Some other authors defended the application of multi-slope models on cellular networks [210,211,212,213,214]. These authors assert extending the log-distance path loss model to a multi-slope variant with multiple distance–power gradients enhances its effectiveness in representing the radio channel and its fluctuations. By considering physical characteristics, such as terrain and obstacles, multi-slope path loss offers a comprehensive approach to modelling signal propagation in diverse environments.

We assessed the multi-slope model using propagation measurements obtained from LoRa systems. To interpret the significance of the log-distance parameters for each region, we have also evaluated the parametrization of propagation results for different systems. Interestingly, some studies have reported path loss exponents considerably lower than those observed in free space scenarios [23,165,212]. For instance, a single-slope model applied to data depicted in Figure 7d gives β=1.4 when the received signal reaches the highest attenuation values for small distances from the transmitter.

**Figure 11:**
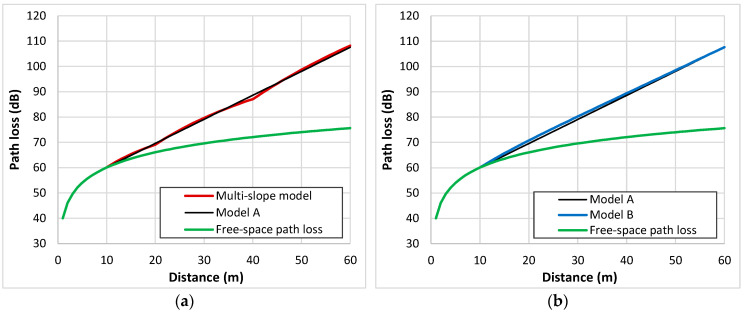
Multi-slope model example: (**a**) model results defined in [210] and linear approximation; (**b**) parameter *β* as a function of *α*.

Let us examine the following example: Andrade and Hoefel [210] describe a log-distance model for indoor environments featuring four distinct regions. The parameters of Equation (2) defining this multi-slope model are as follows: α=40.05, β=2 for 1<d≤10; α=30.05, β=3 for 10<d≤20; α=−8.95, β=6 for 20<d≤40; α=−104.95, β=12 for d>40. Figure 11a displays the results for a range from 1 to 60 m. Initially, the model follows the free-space behaviour up to 10 m, transitioning thereafter into an almost linear trend. The high value of β=12 appears elevated compared to the empirical findings. To address this issue, we approximated the region above 10 m by a linear tendency as a function of distance, defined as model A. Here, α=50.58 and β increases from 1 at 11 m to 3.2 at 60 m. However, such low β values, diverging significantly from the free-space path loss exponent, may not accurately represent real-world scenarios. Alternatively, we propose a linear increase in β above 2 for distances exceeding 10 m as a more plausible interpretation. Figure 11b illustrates the results, with α=40.05 (reflecting free-space path loss at 1 m) and β progressively rising from 2 at 10 m to 3.8 at 60 m (model B).

**Figure 12:**
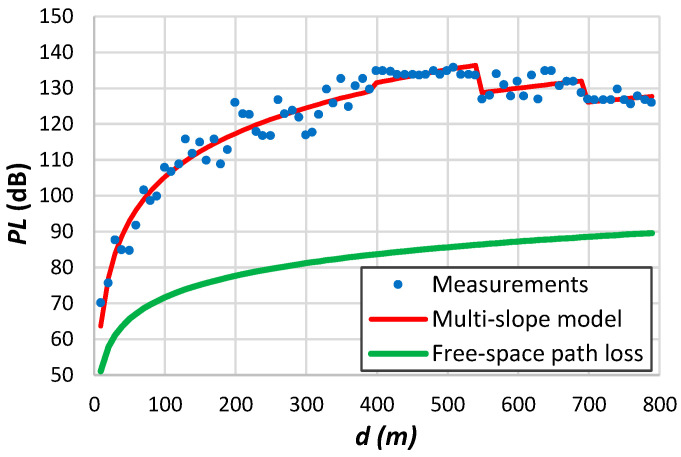
Multi-slope model applied to data represented in Figure 7b.

The single-slope model parameters corresponding to the data depicted in Figure 7b are in the twelfth row of Table 12. The application of a multi-slope model segmented into four regions to this dataset, as illustrated in Figure 12, enhanced the RMSE from 5.39 dB to 3.26 dB. However, careful consideration was necessary in extracting the log-distance parameters. For instance, during curve-fitting for the second region, α=137.1 and β=−0.11 were obtained, indicating an unrealistic β value. Incorporating the initial data point, situated approximately 10 m from the transmitter, yielded α=34.36 and β=3.73. This process emphasizes the importance of considering data points near the transmitter to characterize the model’s behaviour.

**Figure 13:**
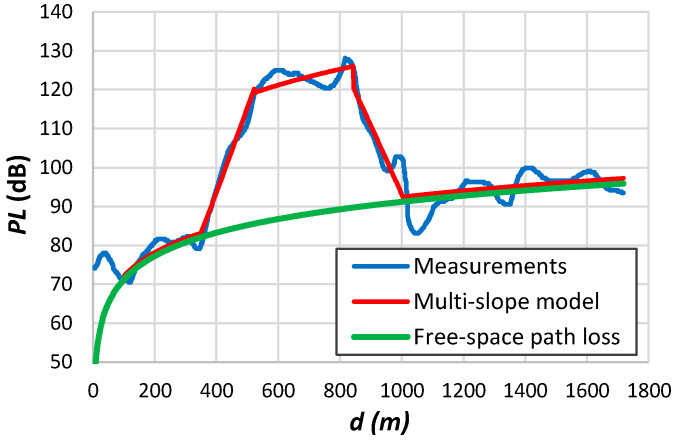
Multi-slope model applied to data outlined in [79].

Figure 13 represents a scenario where the path loss conforms to free space propagation in two regions and has an additional zone characterized by higher values [79]. Utilizing the single-slope model yields an RMSE of 13.97 dB. We employed the multi-slope model, assigning β=2 to the regions exhibiting path loss similar to free-space propagation and β=3.26 to the zone experiencing higher path loss. Between zones, β increased or decreased linearly with distance. This model reduced the RMSE to 4.56 dB. As can also be seen in Figure 13, the path loss is high for short distances from the transmitter. This discrepancy may be due to the significant difference in transmitter and receiver antenna heights. Excluding these distances, the RMSE was reduced to 3.10 dB.

**Figure 14:**
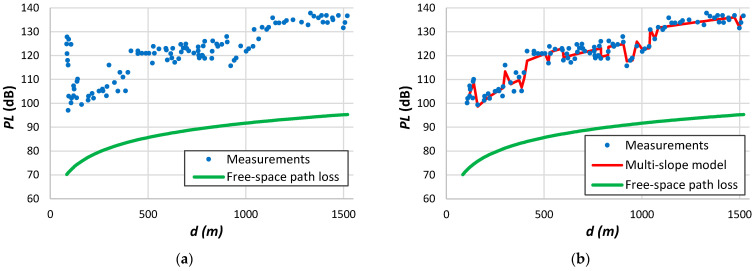
Data measured in an urban route: (**a**) measurements; (**b**) multi-slope model.

Several authors measured path loss across extensive areas within an environment, grouping the data based on distance from the transmitter. In such cases, it becomes challenging to associate the measurements with their acquisition locations. However, in certain instances, authors have depicted data acquired along specific routes within an environment. Figure 14a presents an illustrative example of this approach [28]. By explicitly stating the location of the measurement campaign, the article facilitated the examination of urban environment variations using the ‘Street View’ feature of Google Maps. Upon inspecting Figure 14a, a distinct zone with notably high path loss values around the transmitter becomes apparent. Modelling the path loss within this zone requires knowledge of the measurement setup geometry not presented in the paper. Utilizing the details of a 50 m gateway height, a terminal antenna height of 1.8 m, and the distance to the transmitter, we applied the knife-edge method to estimate the diffraction loss caused by the building edge. Incorporating an estimation for loss attributable to the antenna radiation pattern, we derived an excess attenuation ranging from 20 to 30 dB around the transmitter. These estimates align with the values depicted in Figure 14a. Upon removing this zone, a single-slope model yielded an RMSE of 4.23 dB. Although the path loss above the previous zone diminishes, it maintains high values. The log-distance parameters were determined to be α=−19.18 and β=5.94, indicating a substantial influence of building diffraction on path loss. Above this zone, we identified four distinct groups of path loss, characterized by the following log-distance parameters pairs: (α,β) = (31.67, 2.90), (31.96, 3.02), (31.80, 3.15), and (31.90, 3.29). Figure 14b illustrates these results. The application of a multi-slope model reduced the RMSE to 1.84 dB.

**Figure 15:**
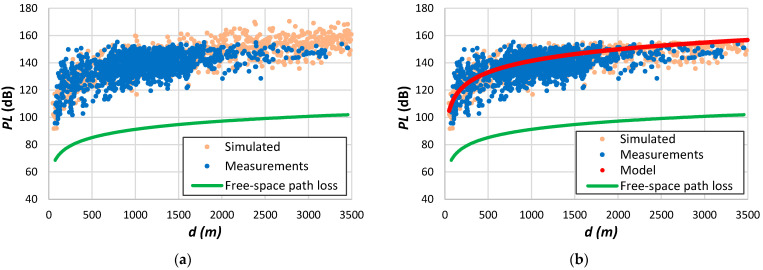
Data with a great transitional region: (**a**) measurements and simulated results; (**b**) after elimination of simulated values above a threshold.

In specific scenarios, employing a multi-slope model instead of a single-slope model may lead to incorrect outcomes. For example, in Figure 15a, the blue dots represent data collected from Liu et al. [65]. Above 700 m, the path loss reaches a peak due to the receiver sensitivity (155 dB). The log-distance model parameters are shown in Table 12. Observing a distinct slope in the transitional region, some researchers have attempted to model it using different parameters. To delve into this matter, we utilized the α and β values acquired below the transitional region to simulate path loss values. Random values, with a standard deviation derived from the RMSE, were added to the log-distance model, resulting in the simulated outcomes depicted in Figure 15a. Filtering out simulated values above 155 dB, we obtained the results illustrated in Figure 15b. As we may observe, these data closely match the measured values, reflecting the impact of packet loss on path loss in LoRa systems.

## 6. Discussion and Conclusions

The categorization of propagation models often lacks uniformity among different researchers. Based on this review, we identified three primary propagation models: empirical, deterministic, and active measurements. Employing deterministic models for network planning in LoRa systems poses challenges owing to the necessity for extensive databases that encompass environmental parameters. While these models might find utility in areas with limited spatial extents, the long-range achievable with LoRa makes their application impractical. Consequently, the prevailing approach in the literature favours empirical or active measurement models for estimating path loss in diverse propagation environments. Active measurement models require path loss measurements to be conducted within the specific environment where the model will be applied. On the other hand, empirical models should incorporate parameters designed to adapt to diverse propagation environments.

From this study, we concluded that the most used empirical propagation model to describe path loss considers the logarithmic distance decay. The log-distance model based on the close-in form employs the parameter $PL\left(d_{0}\right),$ defining the free-space path loss at a reference distance $d_{0}.$ Researchers have imposed this reference typically at 1 m, 100 m, or 1 km. By fixing this parameter, the model only requires the path loss exponent. However, based on actual measurements, there is no justification for assuming a reference distance of 1 km. The second form for the log-distance model, as described by Equation (2), incorporates two parameters. The first parameter, α, denotes the path loss at a distance of one meter, while the second parameter, β, represents the slope of the trend line concerning distance. Applying this model to the actual propagation measurements has enabled the determination of the reference distance. Additionally, the log-normal model includes a third parameter, $X_{\sigma }$, a zero-mean Gaussian distributed random variable accounting for the fading effect. This parameter adds further information to the model, describing the stochastic nature of wireless propagation phenomena.

Upon examining various empirical models for outdoor environments, we found that most of them could be characterized using a log-distance model. Even models exhibiting linear variations with distance are log-distance, with β increasing over distance. By employing the floating-intercept model, we can effectively determine the α and β parameters, which correlate with environmental characteristics and communication system parameters. The categorization into different terms, as proposed in our work, offers insight into the influence of environmental parameters on path loss. For instance, let us consider the impact of frequency among outdoor models. Using the frequency of 870 MHz typical in LoRa applications for urban environments, we observed a wide range of values for $P_{1}\left(f\right)$ ranging from −39 dB to 41 dB, with an average of 12.5 dB and a standard deviation of 20.6 dB. This dispersion of results prompts us to question whether the proposed models adequately account for the frequency effect on path loss. Many of these models only consider the distance to the transmitter, the carrier frequency, and antenna heights as input parameters. Some models incorporate additional environmental parameters primarily relevant to urban environments, such as average building height, distance between buildings, street width, and angle of arrival relative to the street axis. These parameters reflect the complexity of urban propagation environments and highlight the need for more comprehensive models for a broader range of factors.

For empirical models applied to indoor environments, we could again represent them using a log-distance model. Typically, the input parameters from the environment include the number of walls in the propagation path, the wall attenuation, the number of floors in the propagation path, and the floor attenuation. These parameters collectively define α, reflecting the initial path loss at close distances. While several models incorporate the β parameter of free space, others necessitate actual measurements to ascertain β, capturing the rate of path loss increase with distance within the indoor environment.

Empirical models designed for environments within vegetation initially considered excess attenuation above free-space loss rather than path loss. Consequently, several models deviate from the log-distance model framework. However, upon analyzing measurements used to develop some of these models, it became evident that the error incurred by substituting those models with a log-distance model is negligible. Thus, we concluded that log-distance models could effectively replace these alternative models. Some authors have proposed log-distance models adapted for environments within vegetation. The parameters employed in these models are tree density, average trunk diameter, number of trees, average tree loss, leaf dimensions, leaf density, height of trees without leaves, and canopy diameter. By including these parameters, empirical models offer a more accurate approach to modelling propagation within vegetation environments.

Active measurement models use empirical data from field measurements to characterize radio wave propagation environments. These models can incorporate parameters such as path loss, shadowing, fading, and multipath effects, offering a robust framework for assessing signal propagation. Machine learning models offer advantages over curve-fitting models by learning patterns directly from data, outperforming empirical models in some scenarios and requiring less feature engineering. Hybrid approaches combining machine learning techniques with traditional propagation models or curve-fitting methods leverage the strengths of both approaches, improving prediction accuracy. As a result, active measurement-based propagation models offer substantial advantages for LoRa network planning and optimization, providing more accurate predictions than empirical models and facilitating validation and refinement of other models. The problem associated with active measurement propagation models is the necessity to perform measurements in the environments, mainly for LoRa systems because of their long range. However, we can use these procedures to obtain the main parameters employed in empirical models and to determine how they affect path loss.

Based on our evaluation of the measurement results from published works, we have concluded that the reference distance $d_{0}$ exhibits a notable increase with parameter β. Furthermore, we found that the highest values of $d_{0}$ observed outdoors were below 80 m. Conversely, indoor environments yielded lower reference distances, even for large values of β, as measurement distances rarely exceeded a few tens of meters. We also observed these findings in results within vegetation environments. Therefore, we have concluded that $d_{0}$ should not be constant but a function of β. Our analysis revealed an inverse relationship between α and β, too. This relationship was approximately linear for outdoor environments, where more data were available. Considering the correlation between α and β, our study suggests that specifying one parameter while deriving the other through a trend line could offer a more simplified approach to developing new models.

Our study also evaluated LoRa measurements, revealing distinctive characteristics compared to other communication systems. In contrast to many systems operating with values of SNR above zero, LoRa can operate even with negative SNR values. This peculiarity makes RSSI inadequate to serve as the right measure of the received signal quality. Below an SNR of 0 dB, the RSSI becomes primarily influenced by channel noise and interference, often plateauing at a minimum value. In this case, we determined the ESP, offering a more accurate measure of the received signal. Another situation found in the literature and experiments is that the RSSI provided by LoRa radios can have high errors, with discrepancies sometimes reaching up to 20 dB. These errors introduce considerable inaccuracies in path loss estimations, requiring the calibration of LoRa radios to rectify these discrepancies. The LoRa measurements documented in published works corroborate several findings from other communication systems. However, a notable distinction lies in the substantial transitional region observed in LoRa measurements, significantly affecting the development of propagation models tailored to LoRa environments. We observed deriving log-distance parameters using data below this transitional region is essential for accurate modelling. Moreover, if the environmental characteristics remain consistent, analyzing received sensitivity and the standard deviation of path loss could offer insights into packet error rates. Nevertheless, more studies are required to validate this assumption.

Upon confronting existing models against LoRa measurements for outdoor environments, we concluded that most of these models yield limited results. The range of outcomes fails to align closely with the actual data, thus rendering them inadequate for accurate predictions. Consequently, employing such models leads to significant prediction errors in various scenarios. Notably, only models incorporating parameters specific to the environment demonstrate better alignment with the observed data, a trend reinforced by some results obtained within vegetated environments. Moreover, after observation of the poor outcomes reached with existing empirical models, some authors have resorted to tuning these models to enhance prediction accuracy [33,35,47,54]. However, given that most empirical models are log-distance, there is no need to tune model constants. Instead, such adjustment is an indirect means of determining the α and β parameters of the log-distance model.

Our observations also underscored the imperative for a multi-slope model instead of a single-slope, as revealed by numerous measurement results. Given the extensive coverage range of LoRa systems, keeping log-distance parameters constant across all positions within a given environment becomes unreal. Consequently, the development and implementation of a multi-slope model becomes essential. We found that employing such a modelling procedure reduces the prediction errors, highlighting its efficacy in capturing the nuanced variations in signal propagation across different locations within the same region.

Modelling path loss propagation in a given environment involves determining the three parameters of the log-normal model. However, various authors developed empirical models using measurements aggregating data acquired in large ranges, posing challenges for accurate model extraction. For instance, measurements acquired along a specific route can facilitate the modelling process by providing more cohesive data. Empirical models should incorporate environmental parameters to ensure applicability across different regions. Machine learning techniques offer a promising avenue for evaluating the connection between model parameters and environmental factors due to the ability to learn non-linear relationships. Such approaches could lead to developing models that are more tuned to the environment.

It is important to note that the measurements should include the third parameter of the log-normal model, $X_{\sigma }$, which is essential for assessing the fading effect. For example, investigating potential relationships between α, β, and $X_{\sigma }$ parameters, analogous to the correlation observed between α, β, and $d_{0}$, can provide valuable insights. Small-scale fading phenomena are inherently specific to each position in the environment, particularly evident in measurements acquired within a few wavelengths. Large-scale fading accounts for signal variation over large distances. Future advancements in propagation modelling could benefit from considering the log-distance model as the foundation for new empirical models. Furthermore, new measurement campaigns with LoRa systems should explore innovative approaches to data utilization. Ultimately, research with new measurement data should aim to uncover correlations between model parameters and environmental factors, elucidating the intricate relationship between the two domains and advancing our understanding of propagation characteristics.

## Figures and Tables

**Table 1:** Parameters for the Egli and Lee models.

Model	Component of *α*	Value (dB)	Component of *β*	Value (dB)
Egli model	$K_{\alpha }$	−16.14	$K_{\beta }$	4
	$P_{1}\left(f\right)$	-	$F_{1}\left(f\right)$	-
	$P_{2}\left(h_{B},h_{T}\right)$	$-20{log}_{10}\left(h_{B}\right)-10{log}_{10}\left(h_{T}\right),\;\;h_{T}\leq 10\;m$	$F_{2}\left(h_{B},h_{T}\right)$	-
Lee Model	$K_{\alpha }$	$\left\{\begin{array}{c} L_{0}+72.80-10m{log}_{10}\left(900\right)-G_{B}+G_{T}-3\gamma ,h_{T}\geq 3\\ L_{0}+68.03-10m{log}_{10}\left(900\right)-G_{B}+G_{T}-3\gamma ,h_{T}<3 \end{array}\right.$	$K_{\beta }$	$\frac{\gamma }{10}$
	$P_{1}\left(f\right)$	$(10m-20){log}_{10}\left(f\right)$	$F_{1}\left(f\right)$	-
	$P_{2}\left(h_{B},h_{T}\right)$	$\left\{\begin{array}{cc} -20{log}_{10}\left(h_{B}\right)-20{log}_{10}\left(h_{T}\right), & h_{T}\geq 3\\ -20{log}_{10}\left(h_{B}\right)-10{log}_{10}\left(h_{T}\right), & h_{T}<3 \end{array}\right.$	$F_{2}\left(h_{B},h_{T}\right)$	-

**Table 2:** Reference parameters for the Lee model.

Environment	$L_{0}$ (dB)	γ
Open (rural area)	89	43.5
Suburban	102	38.5
Urban—Philadelphia	110	36.8
Urban—Newark	104	43.1
Urban—Tokyo	124	30.5

**Table 3:** Parameters for the Okumura–Hata model.

Environment	Component of *α*	Value (dB)	Component of *β*	Value (dB)
Urban: large cities	$K_{\alpha }$	−36.29	$K_{\beta }$	4.49
	$P_{1}\left(f\right)$	$6.16{log}_{10}\left(f\right)$	$F_{1}\left(f\right)$	-
	$P_{2}\left(h_{B},h_{T}\right)$	$5.83{log}_{10}\left(h_{B}\right)-3.2{\left[{log}_{10}\left(h_{T}\right)\right]}^{2}-2.14{log}_{10}\left(h_{T}\right)$	$F_{2}\left(h_{B},h_{T}\right)$	$-0.655{log}_{10}\left(h_{B}\right)$
Urban: medium–small cities	$K_{\alpha }$	−38.39	$K_{\beta }$	4.49
	$P_{1}\left(f\right)$	$7.72{log}_{10}\left(f\right)$	$F_{1}\left(f\right)$	-
	$P_{2}\left(h_{B},h_{T}\right)$	$5.83{log}_{10}\left(h_{B}\right)-\left[1.1{log}_{10}\left(f\right)-0.7\right]h_{T}$	$F_{2}\left(h_{B},h_{T}\right)$	$-0.655{log}_{10}\left(h_{B}\right)$
Suburban/rural	$K_{\alpha }$	−47.98	$K_{\beta }$	4.49
	$P_{1}\left(f\right)$	$13.51{log}_{10}\left(f\right)-2{\left[{log}_{10}\left(f\right)\right]}^{2}$	$F_{1}\left(f\right)$	-
	$P_{2}\left(h_{B},h_{T}\right)$	$5.83{log}_{10}\left(h_{B}\right)-\left[1.1{log}_{10}\left(f\right)-0.7\right]h_{T}$	$F_{2}\left(h_{B},h_{T}\right)$	$-0.655{log}_{10}\left(h_{B}\right)$
Rural	$K_{\alpha }$	−79.33	$K_{\beta }$	4.49
	$P_{1}\left(f\right)$	$26.051{log}_{10}\left(f\right)-4.78{\left[{log}_{10}\left(f\right)\right]}^{2}$	$F_{1}\left(f\right)$	-
	$P_{2}\left(h_{B},h_{T}\right)$	$5.83{log}_{10}\left(h_{B}\right)-\left[1.1{log}_{10}\left(f\right)-0.7\right]h_{T}$	$F_{2}\left(h_{B},h_{T}\right)$	$-0.655{log}_{10}\left(h_{B}\right)$

**Table 4:** Parameters for Okumura–Hata extension models.

Model Environment	Component of *α*	Value (dB)	Component of *β*	Value (dB)
Ericson model Urban	$K_{\alpha }$	$a_{0}-30a_{1}+23.89$	$K_{\beta }$	${0.1a}_{1}$
	$P_{1}\left(f\right)$	$24.49{log}_{10}\left(f\right)-4.78{\left[{log}_{10}\left(f\right)\right]}^{2}$	$F_{1}\left(f\right)$	-
	$P_{2}\left(h_{B},h_{T}\right)$	$(a_{2}-30a_{3}){log}_{10}\left(h_{B}\right)-3.2{\left[{log}_{10}\left(h_{T}\right)\right]}^{2}-6.85{log}_{10}\left(h_{T}\right)$	$F_{2}\left(h_{B},h_{T}\right)$	$0.1a_{3}{log}_{10}\left(h_{B}\right)$
COST 231-Hata Urban	$K_{\alpha }$	−56.54	$K_{\beta }$	4.49
	$P_{1}\left(f\right)$	$13.9{log}_{10}\left(f\right)$	$F_{1}\left(f\right)$	-
	$P_{2}\left(h_{B},h_{T}\right)$	$5.83{log}_{10}\left(h_{B}\right)-3.2{\left[{log}_{10}\left(h_{T}\right)\right]}^{2}-6.85{log}_{10}\left(h_{T}\right)$	$F_{2}\left(h_{B},h_{T}\right)$	$-0.655{log}_{10}\left(h_{B}\right)$
COST 231-Hata Suburban/Rural	$K_{\alpha }$	−61.64	$K_{\beta }$	4.49
	$P_{1}\left(f\right)$	$15.46{log}_{10}\left(f\right)$	$F_{1}\left(f\right)$	-
	$P_{2}\left(h_{B},h_{T}\right)$	$5.83{log}_{10}\left(h_{B}\right)-\left[1.1{log}_{10}\left(f\right)-0.7\right]h_{T}$	$F_{2}\left(h_{B},h_{T}\right)$	$-0.655{log}_{10}\left(h_{B}\right)$
SUI model Type A	$K_{\alpha }$	−68.56	$K_{\beta }$	4.6
	$P_{1}\left(f\right)$	$6{log}_{10}\left(f\right)$	$F_{1}\left(f\right)$	-
	$P_{2}\left(h_{B},h_{T}\right)$	$0.15h_{B}-\frac{252}{h_{B}}-10.8{log}_{10}\left(h_{T}\right)$	$F_{2}\left(h_{B},h_{T}\right)$	$\frac{12.6}{h_{B}}-0.0075h_{B}$
SUI model Type B	$K_{\alpha }$	−56.56	$K_{\beta }$	4.0
	$P_{1}\left(f\right)$	$6{log}_{10}\left(f\right)$	$F_{1}\left(f\right)$	-
	$P_{2}\left(h_{B},h_{T}\right)$	$0.13h_{B}-\frac{342}{h_{B}}-10.8{log}_{10}\left(h_{T}\right)$	$F_{2}\left(h_{B},h_{T}\right)$	$\frac{17.1}{h_{B}}-0.0065h_{B}$
SUI model Type C	$K_{\alpha }$	−45.79	$K_{\beta }$	3.6
	$P_{1}\left(f\right)$	$6{log}_{10}\left(f\right)$	$F_{1}\left(f\right)$	-
	$P_{2}\left(h_{B},h_{T}\right)$	$0.1h_{B}-\frac{400}{h_{B}}-20{log}_{10}\left(h_{T}\right)$	$F_{2}\left(h_{B},h_{T}\right)$	$\frac{20}{h_{B}}-0.005h_{B}$
ECC-33 model Urban	$K_{\alpha }$	206.33	$K_{\beta }$	2.98
	$P_{1}\left(f\right)$	$9.56{\left[{log}_{10}\left(f\right)\right]}^{2}-41.45{log}_{10}\left(f\right)$	$F_{1}\left(f\right)$	-
	$P_{2}\left(h_{B},h_{T}\right)$	$-66.16{log}_{10}\left(h_{B}\right)-\left[42.57+13.7{log}_{10}\left(\frac{f}{1000}\right)\right]{log}_{10}\left(h_{T}\right)$	$F_{2}\left(h_{B},h_{T}\right)$	$0.58\left[{6-log}_{10}\left(d\right)\right]{log}_{10}\left(\frac{h_{B}}{200}\right)$

**Table 5:** Parameters for models that consider environmental characteristics.

Model	Component of *α*	Value (dB)	Component of *β*	Value (dB)
Edwards–Durkin model	$K_{\alpha }$	27.56	$K_{\beta }$	4
	$P_{1}\left(f\right)$	$-20{log}_{10}\left(f\right)$	$F_{1}\left(f\right)$	-
	$P_{2}\left(h_{B},h_{T}\right)$	$-20{log}_{10}\left(h_{B}\right)-20{log}_{10}\left(h_{T}\right)$	$F_{2}\left(h_{B},h_{T}\right)$	-
	$P_{3}\left(\upsilon \right)$	$L_{D}$	$F_{3}\left(\upsilon \right)$	-
Ikegami model	$K_{\alpha }$	−8.19	$K_{\beta }$	2
	$P_{1}\left(f\right)$	$10{log}_{10}\left(f\right)$	$F_{1}\left(f\right)$	-
	$P_{2}\left(h_{B},h_{T},H\right)$	$20{log}_{10}\left({H-h}_{T}\right)$	$F_{2}\left(h_{B},h_{T},H\right)$	-
	$P_{3}\left(W,\phi \right)$	− $10{log}_{10}\left(W\right)$ $\;+\;10{log}_{10}\left[sin(\phi )\right]$	$F_{3}\left(W,\phi \right)$	-
Walfisch–Bertoni model	$K_{\alpha }$	3.1	$K_{\beta }$	3.8
	$P_{1}\left(f\right)$	${log}_{10}\left(f\right)$	$F_{1}\left(f\right)$	-
	$P_{2}\left(h_{B},h_{T},h\right)$	$-18{log}_{10}\left(h_{B}-h\right)$	$F_{2}\left(h_{B},h_{T},h\right)$	-
	$P_{3}\left(D\right)$	$5{log}_{10}\left[\frac{D^{2}}{4}+{\left({h-h}_{T}\right)}^{2}\right]-5{log}_{10}\left(D\right)$ $+20{log}_{10}\left[atan\left(2\frac{{h-h}_{T}}{D}\right)\right]$	$F_{3}\left(D\right)$	-
COST 231-Walfisch–Ikegami model	$K_{\alpha }$	$37.1-{3k}_{d}$	$K_{\beta }$	3.8
	$P_{1}\left(f\right)$	$(10+k_{f}){log}_{10}\left(f\right)$	$F_{1}\left(f\right)$	-
	$P_{2}\left(h_{B},h_{T},h\right)$	$L_{bsh}+20{log}_{10}\left({h-h}_{T}\right)$ $-\left\{\begin{array}{c} 0.8\left(h_{B}-h\right){,h}_{B}\leq h\;and\;d>500\\ 0,others \end{array}\right.$	$F_{2}\left(h_{B},h_{T},h\right)$	$L_{a}+\left\{\begin{array}{c} -1.5\left(\frac{h_{B}-h}{h}\right){,h}_{B}\leq h\\ 0,others \end{array}\right.$
	$P_{3}\left(W,\phi \right)$	− $10{log}_{10}\left(W\right)+L_{ori}$	$F_{3}\left(W,\phi \right)$	-
	$P_{4}\left(D\right)$	− $9{log}_{10}\left(D\right)$	$F_{3}\left(D\right)$	-
3GPP model LOS Rural	$K_{\alpha }$	-	$K_{\beta }$	$\left\{\begin{array}{cc} 2 & ,\;d<d_{BP}\\ 4 & ,\;d\geq d_{BP} \end{array}\right.$
	$P_{1}\left(f\right)$	-	$F_{1}\left(f\right)$	-
	$P_{2}\left(h_{B},h_{T},h\right)$	$-min\left(0.04{4h}^{1.72},14.77\right),{\;d<d}_{BP}$ $-20{log}_{10}\left(d_{BP}\right)-min\left(0.044h^{1.72},14.77\right)$ $+min\left(0.03h^{1.72},10\right){log}_{10}\left(d_{BP}\right)+$ $0.002{log}_{10}\left(h\right)d_{BP},\;\;d\geq d_{BP}$	$F_{2}\left(h_{B},h_{T},h\right)$	$\min\left(0.003h^{1.72},1\right)$ $+0.0002\frac{{log}_{10}(h)d}{{log}_{10}(d)},d<d_{BP}$ $0,\;\;\;d\geq d_{BP}$
3GPP model NLOS Rural	$K_{\alpha }$	−0.36	$K_{\beta }$	4.34
	$P_{1}\left(f\right)$	-	$F_{1}\left(f\right)$	-
	$P_{2}\left(h_{B},h_{T},h\right)$	$-\left[15.07-3.7{\left(\frac{h}{h_{B}}\right)}^{2}\right]{log}_{10}\left(h_{B}\right)$ $+7.5{log}_{10}\left(h\right)-3.2{\left[{log}_{10}\left(h_{T}\right)\right]}^{2}$ $-6.85{log}_{10}\left(h_{T}\right)$	$F_{2}\left(h_{B},h_{T},h\right)$	${-0.31log}_{10}\left(h_{B}\right)$
	$P_{3}\left(W\right)$	$-7.1{log}_{10}\left(W\right)$	$F_{3}\left(W\right)$	-

**Table 6:** Parameters for indoor models.

Model	Component of *α*	Value (dB)	Component of *β*	Value (dB)
Motley–Keenan model [113]	$K_{\alpha }$	-	$K_{\beta }$	2
	$P_{2}\left(\alpha_{F},\alpha_{W}\right)$	$n_{F}\alpha_{F}+n_{W}\alpha_{W}$	$F_{2}\left(\alpha_{F},\alpha_{W}\right)$	-
Seidel–Rappaport model [92]	$K_{\alpha }$	$(20-10n){log}_{10}\left(d_{0}\right)$	$K_{\beta }$	n
	$P_{2}\left(FAF\right)$	FAF	$F_{2}\left(FAF\right)$	-
COST 231 multi-wall model [102]	$K_{\alpha }$	$L_{C}$	$K_{\beta }$	2
	$P_{2}\left(\alpha_{F},\alpha_{W}\right)$	${n_{F}}^{\left(\frac{n_{F}+2}{n_{F}+1}-b\right)}\alpha_{F}+\sum_{i=1}^{I}n_{Wi}\alpha_{Wi}$	$F_{2}\left(\alpha_{F},\alpha_{W}\right)$	-
IDP model [115,116]	$K_{\alpha }$	-	$K_{\beta }$	2
	$P_{2}\left(\alpha_{F},\alpha_{W}\right)$	$\sum_{i=1}^{I}L_{Wi}+\sum_{j=1}^{J}L_{Bj}$	$F_{2}\left(\alpha_{F},\alpha_{W}\right)$	-
Tuan et al. model [117]	$K_{\alpha }$	$k_{1}+G_{a}-27.56$	$K_{\beta }$	$k_{3}$
	$P_{1}\left(f\right)$	${(k}_{2}-20){log}_{10}\left(f\right)$	$F_{1}\left(f\right)$	-
	$P_{2}\left(n_{W},n_{F},P_{1},P_{2}\right)$	${n_{W}{(k}_{4}P_{1}{+k}_{5}P_{2})+k}_{6}n_{F}$	$F_{2}\left(n_{W},n_{F},P_{1},P_{2}\right)$	-
Barbosa et al. model [118]	$K_{\alpha }$	-	$K_{\beta }$	-
	$P_{2}\left(n_{F},a,b\right)$	$\frac{a+\frac{abn_{F}}{2}+\frac{ab^{2}n_{F}^{2}}{12}}{1-\frac{bn_{F}}{2}+\frac{b^{2}n_{F}^{2}}{12}}$	$F_{2}\left(n_{F},a,b\right)$	$-0.109n_{F}^{2}+0.853n_{F}+1.51$
Degli-Esposti et al. model [119]	$K_{\alpha }$	$(20-10n){log}_{10}\left(d_{0}\right)$	$K_{\beta }$	n
	$P_{2}\left(\gamma \right)$	-	$F_{2}\left(\gamma \right)$	$\gamma \frac{d}{{log}_{10}\left(d\right)},\;d>1$

**Table 7:** Excess attenuation models for environments within vegetation.

Model	*L* (dB)
Modified Exponential Model [122]	$\left\{\begin{array}{cc} 0.063f^{0.284}d & ,\;d\leq 14\\ 0.187f^{0.284}d^{0.588} & ,\;14<d\leq 400 \end{array}\right.$
ITU-R [123]	$0.2f^{0.3}d^{0.6}$
COST 235 [124]	$\left\{\begin{array}{cc} 15.6f^{-0.009}d^{0.26} & ,in-leaf\\ 26.6f^{-0.2}d^{0.5} & ,out-of-leaf \end{array}\right.$
FITU-R [125]	$\left\{\begin{array}{cc} 0.39f^{0.39}d^{0.25} & ,in-leaf\\ 0.37f^{0.18}d^{0.59} & ,out-of-leaf \end{array}\right.$
Chen-Kuo model [126]	$\left\{\begin{array}{c} \left(f\times 10^{-6}+0.2\right)d+5f\times 10^{-4}+3,\;vertical\\ \left(2f\times 10^{-7}+0.2\right)d+3f\times 10^{-5}+2,\;horizontal \end{array}\right.$
LITU-R [127]	$0.48f^{0.43}d^{0.13}$

**Table 9:** Parameters derived from outdoor measurement data found in published works.

Reference	Environment	Range (m)	*f* (MHz)	*α* (dB)	*β*	RMSE	$d_{0}$ (m)	*PL_FS_* (1 m)	*n*	RMSE_1_
[163]	Urban	180–8000	203	25.25	2.89	9.46	0.18	18.60	3.08	9.48
[164]	Urban	100–2000	800	42.23	2.85	2.56	0.04	30.50	3.25	2.89
[161]	Urban	10–1100	811	81.01	1.77	11.36	-	30.62	3.69	13.08
[111]	Urban	400–16,000	820	46.58	2.54	5.62	0.001	30.72	2.96	5.88
[111]	Urban	300–15,000	820	17.80	3.57	4.49	6.67	30.72	3.21	4.72
[165]	Urban	100–2000	900	−44.96	6.26	7.34	62.7	31.53	3.53	11.61
[166]	Urban	200–3000	900	55.87	2.81	4.88	0.001	31.53	3.58	5.41
[167]	Urban	60–900	1800	88.84	1.45	7.36	-	37.55	3.36	9.32
[168]	Suburban	400–1400	1800	−10.41	5.06	3.76	37.0	37.55	3.43	4.72
[169]	Suburban	300–3800	1800	23.89	3.27	3.93	11.9	37.55	2.85	4.09
[170]	Urban	5–4000	1800	64.48	2.45	11.1	10^−6^	37.55	3.28	11.96
[171]	Urban	70–1300	2000	24.06	3.35	4.13	11.7	38.46	2.81	4.57
[172]	Urban	40–900	2100	22.40	3.53	3.02	11.9	38.89	2.90	3.65
[173]	Urban	100–1400	2200	47.39	2.56	4.92	0.04	39.29	2.85	4.98
[153]	Urban	60–1100	2500	34.79	3.78	8.20	2.1	40.40	3.57	8.22
[174]	Rural	400–3300	3500	54.44	2.12	10.64	6 × 10^−10^	43.32	2.46	10.66
[174]	Suburban	600–6000	3500	67.44	1.81	11.09	-	43.32	2.55	11.23
[174]	Urban	400–2500	3500	65.36	2.37	11.9	10^−6^	43.32	3.12	11.98
[175]	Urban	170–1100	3500	49.11	3.21	4.44	0.33	43.32	3.42	4.45
[176]	Suburban	10–600	5800	98.41	1.20	5.98	-	47.71	3.34	9.84

**Table 10:** Parameters derived from indoor measurement data.

Reference	Environment	Range (m)	*f* (MHz)	*α* (dB)	*β*	RMSE	$d_{0}$ (m)	*PL_FS_* (1 m)	*n*	RMSE_1_
[102]	Four floors	5–50	856	32.04	5.29	15.13	0.94	31.09	5.36	15.13
[177]	Office	1–20	858	36.97	2.12	4.69	10^−5^	31.11	2.71	5.12
[92]	Same floor	1–55	914	28.01	3.58	5.48	1.7	31.66	3.31	5.55
[92]	One floor	5–60	914	44.34	3.52	2.47	0.15	31.66	4.42	3.29
[92]	Three floors	10–40	914	2.83	7.73	5.21	3.2	31.66	5.47	5.88
[102]	Four floors	5–50	1800	46.60	5.09	18.28	0.51	37.55	5.80	18.35
[178]	Three floors	3–30	2500	55.7	0.89	4.40	-	40.40	2.38	5.69
[179]	Corridor	1–20	3500	47.23	2.27	2.85	0.03	43.32	2.64	3.20
[180]	Corridor	5–45	5000	57.81	1.82	3.47	-	46.42	2.64	3.83
[181]	Same floor	1–25	5300	42.10	4.25	5.47	1.6	46.93	3.80	6.07

**Table 11:** Parameters derived from measurement within vegetation.

Reference	Environment	Range (m)	*f* (MHz)	*α* (dB)	*β*	RMSE	$d_{0}$ (m)	*PL_FS_* (1 m)	*n*	RMSE_1_
[182]	Forest-hill	1500–65,000	203	23.26	2.87	14.71	0.3	18.60	2.98	14.72
[127]	Forest	5–1000	300	−7.73	4.24	4.31	21.2	21.98	3.10	8.24
[140]	Forest	50–280	605	52.67	2.64	3.95	10^−4^	28.08	3.75	4.59
[134]	Pine	1–120	870	23.02	2.91	4.80	6.8	31.23	2.48	5.11
[134]	Eucalyptus	1–90	870	18.84	3.72	4.06	5.3	31.23	2.96	5.06
[138]	Forest	1–60	870	16.87	4.50	4.88	3.8	31.23	3.53	6.16
[135]	Forest	1–400	900	8.01	3.15	4.07	0.2	31.53	3.00	4.25
[183]	Forest-hill	20–2600	918	−46.62	6.50	7.38	55.0	31.69	3.80	16.84
[126]	Forest	5–100	1000	21.92	3.81	4.51	3.8	32.44	3.18	5.07
[184]	Plane tree	1–110	1300	36.36	2.77	3.39	0.6	34.72	2.87	3.44
[184]	Common lime	1–60	1300	33.45	3.28	6.73	1.3	34.72	3.19	6.74
[132]	Forest	10–300	1800	19.53	3.36	5.51	21.2	37.55	2.49	6.48
[185]	Palm tree	5–50	2100	34.49	3.68	3.33	1.8	38.89	3.38	3.46
[134]	Pine	1–120	2400	34.48	2.87	4.21	4.4	40.05	2.56	4.40
[186]	Plum orchard	1–120	2400	42.44	2.65	3.18	0.4	40.05	2.78	3.21
[137]	Apple orchard	1–30	2400	43.46	3.43	3.04	0.6	40.05	3.71	3.28
[138]	Forest	1–60	2400	21.88	5.16	5.97	3.8	40.05	3.92	7.66
[187]	Forest	1–100	2400	36.93	3.02	5.05	2.0	40.05	2.83	5.11
[188]	Forest	5–40	2400	34.42	3.80	3.78	2.1	40.05	3.38	3.97
[189]	Campus garden	20–110	2400	5.17	4.97	2.95	14.9	40.05	2.80	5.61
[190]	Forest	1–150	2450	40.07	3.70	5.38	1.0	40.23	3.69	5.38

## Data Availability

Data are contained within the article.
